# Integrating RNA-Seq and Metabolomic Perspectives Reveals the Mechanism of Response to Phosphorus Stress of *Potamogeton wrightii*

**DOI:** 10.3390/plants14233556

**Published:** 2025-11-21

**Authors:** Caiyun Pan, Bing Zhou, Ming Tang, Jingan Chen, Haiquan Yang, Xiaorong Xu

**Affiliations:** 1 School of Life Sciences, Guizhou Normal University, Guiyang 550025, China; pancaiyun@gznu.edu.cn (C.P.); zhoubing@gznu.edu.cn (B.Z.); mingtang@gznu.edu.cn (M.T.); 2 Key Laboratory of National Forestry and Grassland Administration on Biodiversity Conservation in Karst Mountainous Areas of Southwestern China, Guizhou Normal University, Guiyang 550025, China; 3 State Key Laboratory of Environmental Geochemistry, Institute of Geochemistry, Chinese Academy of Sciences, Guiyang 550001, China; chenjingan@vip.skleg.cn (J.C.); yanghaiquan@vip.skleg.cn (H.Y.); 4 Guizhou Province Field Scientific Observation and Research Station of Hongfeng Reservoir Ecosystem, Guiyang 551499, China

**Keywords:** *Potamogeton wrightii*, phosphorus, RNA-seq, metabolome, phosphate transporters

## Abstract

Phosphorus is an important nutrient element in aquatic ecosystems, and its concentration directly affects the growth and ecological functions of submerged plants. However, the physiological and molecular mechanisms of *P. wrightii*’s response to phosphorus stress remain unknown. This study investigated the effects of different phosphorus concentration treatments on *P. wrightii* through physiological, RNA-seq, and metabolome analysis methods. The results indicated that phosphorus stress affected plant physiology by reducing chlorophyll content, increasing MDA and H_2_O_2_ accumulation, and activating the antioxidant enzyme system. Multiple phosphorus transporters (PHT, SPX, and PAP) and the transcription factor PHR1 were identified through RNA-seq and RT-qPCR analysis. The glycerol phospholipids represent a decreasing trend after low or high phosphorus stress. Through the combined analysis of RNA-seq and metabolome analysis, the response differences of 6 DAMs and 19 DEGs to the *P. wrightii* Glycerolipid metabolism and Glycerophospholipid metabolism pathways under different phosphorus stresses were revealed. Our results provide a scientific basis and guidance for restoring submerged plants in shallow lakes and for preventing and controlling eutrophication.

## 1. Introduction

Phosphorus (P) is an essential macronutrient for plants, serving as a fundamental component of nucleic acids, phospholipid membranes, and adenosine triphosphate (ATP) [1]. It plays a crucial role in plant growth, development, and overall metabolic regulation [2]. Moreover, phosphorus is involved in diverse physiological processes, including energy transfer, molecular transport, enzymatic reactions, and photosynthesis [3,4].

Maintaining P homeostasis is essential for optimal plant growth and development. Both P deficiency and excess can adversely affect plant physiological processes. Under low P conditions, plants such as *Arabidopsis thaliana* exhibit inhibited vegetative growth, reduced photosynthetic activity, increased anthocyanin accumulation, and altered root architecture [5,6]. Conversely, high P levels can lead to phosphorus toxicity, characterized by accelerated metabolism, premature development of reproductive organs, suppressed stem and leaf growth, and early termination of vegetative growth [7,8]. Plants respond dynamically to fluctuations in external P availability through a tightly regulated network of metabolic pathways controlled by numerous phosphorus-responsive genes. The expression of these genes varies depending on environmental phosphorus conditions. Previous studies have elucidated the molecular responses of various plant species, including *maize* [9], *soybean* [10], *rice* [11], *wheat* [12], and *Myriophyllum aquaticum* [13], under phosphorus stress.

The phosphorus signaling regulatory network is complex and highly coordinated, involving multiple components, including the phosphate starvation response transcription factor (PHR) family, SPX-domain-containing proteins (SPX family), phosphate transporters (PHT family), microRNAs, and ubiquitination-related proteins [14]. This network plays a critical role in maintaining intracellular and inter-organellar phosphorus balance [15]. Among these regulators, PHR proteins serve as central transcriptional activators in the phosphorus starvation response, directly or indirectly controlling the expression of downstream phosphate starvation-induced (*PSI*) genes. SPX-domain-containing proteins function as phosphorus sensors, modulating the activity of PHRs in response to intracellular phosphorus concentrations [16]. In *rice*, for instance, *OsSPX1* and *OsSPX2* interact with PHRs in the nucleus via their SPX domains, thereby inhibiting PHR binding to the P1BS cis-element [15,17]. Furthermore, *OsSPX4* acts as a negative regulator of OsPHR1. Under phosphorus-deficient conditions, *OsSPX4* undergoes degradation through the 26S proteasome pathway, which releases OsPHR2 to the nucleus, subsequently activating the transcription of downstream *PSI* genes [18]. The PHT family plays a pivotal role in the uptake of inorganic phosphorus (Pi) from the soil and its distribution within plant tissues. PHRs can directly bind to P1BS elements in the promoter regions of PHT genes, thereby regulating their transcription and ultimately controlling phosphorus acquisition and transport in plants [19].

With the rapid development of urbanization, industrialization, and agricultural activities worldwide, eutrophication has become a widespread environmental problem affecting many rivers and lakes. Eutrophication occurs when excessive amounts of nutrients, particularly nitrogen and phosphorus, accumulate in water bodies, resulting in degraded water quality and ecological imbalance [20,21]. Among these nutrients, phosphorus plays a critical role, as its concentration strongly influences the growth, physiological performance, and ecological functions of submerged aquatic plants [13]. Over the past few decades, extensive research has focused on strategies to restore and manage eutrophic water bodies [22,23]. These restoration approaches generally fall into three categories: physical, chemical, and bio-ecological methods. Among these, the use of aquatic plants for ecological restoration has emerged as one of the most cost-effective, environmentally friendly, and sustainable solutions [24,25]. Submerged plants introduced into eutrophic water bodies can efficiently absorb large amounts of nitrogen, phosphorus, and other dissolved nutrients, thereby lowering nutrient concentrations and improving overall water quality [21,26]. Moreover, many macrophytes have the capacity to accumulate heavy metals, absorb and settle suspended particles, and suppress algal blooms. Through these processes, they help purify the water, enhance water transparency, and contribute to the stability and resilience of aquatic ecosystems [27,28].

*Potamogeton wrightii*, a perennial submerged plant belonging to the genus *Potamogeton* in the family *Potamogetonaceae*, is widely distributed across China, from northern to southern provinces, including Taiwan, Guangdong, Jiangsu, Anhui, Yunnan, Guizhou, and Sichuan. Its range also extends to parts of West Asia, northwestern and northeastern India, various Southeast Asian countries, and Japan [29,30]. *P. wrightii* holds both ecological and economic value. It has long been used for medicinal purposes, as bait for herbivorous fish, and as a high-quality feed source for pigs and ducks due to its high nutritional content. Its aerial leaves are relatively thick and rich in keratin and wax, with well-developed stomata, strong tolerance to intense light, and high photochemical efficiency [31]. Moreover, *P. wrightii* exhibits strong ecological adaptability, thriving in a wide range of aquatic environments from oligotrophic to eutrophic water bodies. Its roots, stems, and leaves all possess the ability to absorb phosphorus, and the plant plays a crucial role in regulating phosphorus forms in sediments, inhibiting endogenous phosphorus release, and competing with algae for nutrients. These attributes make it highly effective in purifying various types of effluents and valuable for the prevention and control of lake eutrophication [32,33,34].

Currently, research on the molecular mechanisms underlying phosphorus homeostasis has primarily focused on model plants and major food crops [13,35]. However, little is known about how submerged macrophytes, such as *P. wrightii*, respond to different phosphorus stress conditions at the molecular level. To address this knowledge gap, this study integrates transcriptomic and metabolomic analyses to investigate the differentially expressed genes (DEGs), key functional pathways, and metabolic changes involved in phosphorus homeostasis in *P. wrightii* under varying phosphorus concentrations. By examining changes in gene expression, metabolite profiles, and physiological and biochemical indicators, this study aims to reveal the molecular adaptation mechanisms that enable *P. wrightii* to cope with different levels of P stress.

## 2. Results

### 2.1. Effects of Different Phosphorus Concentrations on Plant Phenotypes and Physiological Indicators

#### 2.1.1. Morphological Differences in Plant Leaves

**Figure 1:**
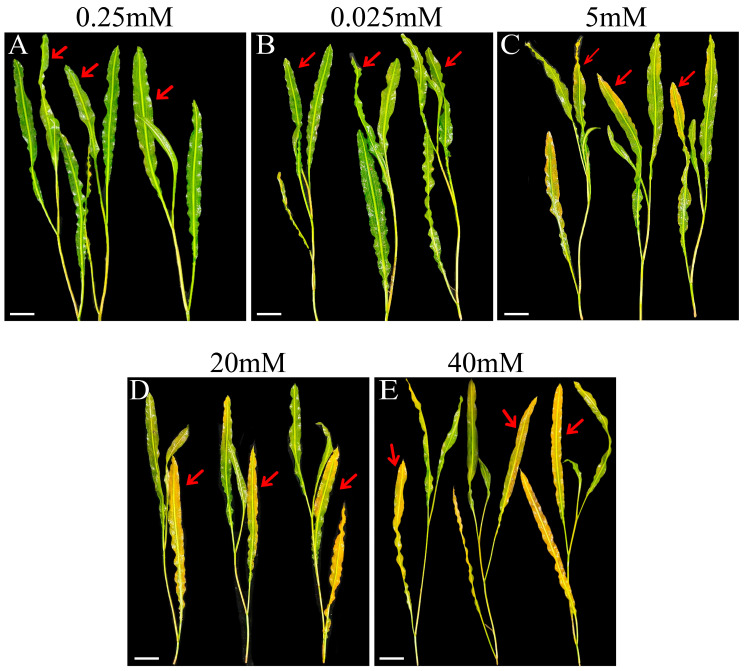
The effect of phosphorus stress on the leaf morphology of *P. wrightii*. Each picture includes three plants, representing three biological replicates respectively. The number at the top of the graph represents the concentration of phosphorus treatment. The red arrows indicate the different shapes of the leaves of plants in different treatment groups. In (**C**–**E**), compared with CK, the leaves indicated by the red arrows are obviously yellowing. (**A**) CK (0.25 mM), (**B**) LP (0.025 mM), (**C**) P5 (5 mM), (**D**) P20 (20 mM), (**E**) P40 (40 mM). Bar = 3 cm.

After 72 h of phosphorus stress treatment, clear morphological changes were observed in the leaves of *P. wrightii* (Figure 1). Compared with the control group (CK), there were no significant differences in leaf morphology under low-phosphorus (LP) conditions. However, in the high-phosphorus (HP) treatment groups (P5, P20, and P40), the leaves exhibited noticeable yellowing. This chlorosis is likely due to HP-induced interference with normal photosynthetic processes, which may have resulted in a reduction in chlorophyll content and subsequent leaf discoloration.

#### 2.1.2. Determination of Chlorophyll Content, Antioxidant Indices, and Inorganic Phosphorus Content

**Figure 2:**
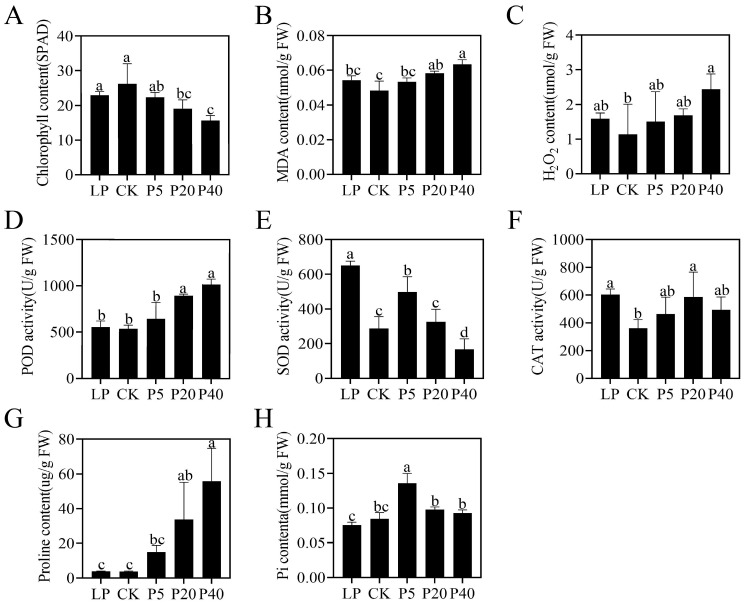
The effects of phosphorus stress on chlorophyll content (**A**), MDA content (**B**), H_2_O_2_ content (**C**), POD activity (**D**), SOD activity (**E**), CAT activity (**F**), proline content (**G**) and Pi content (**H**) in *P. wrightii*. CK (0.25 mM), LP (0.025 mM), HP (5 mM, 20 mM, 40 mM). The different letters in the figure indicate significant differences among the various groups (*p* < 0.05), and the largest mean was marked with a. The meaning was compared with each of the following means, and those not significantly different were marked with a, until an average was found that was significantly different from it, marked b. Take the maximum average number of marks b as the standard and compare it with the following average number of non-marks, where b is not significant, and c is significant. Take the maximum average number of marks c as the criterion and compare it with the following average number of non-marks, where c is not significant, and d is significant. If two samples have the same letter, the difference is not significant.

The chlorophyll content of *P. wrightii* was measured under different phosphorus treatments, and the results are presented in Figure 2A. Compared with the CK treatment group, the P20 and P40 treatment groups showed significant reductions in chlorophyll content, with the P40 treatment group exhibiting the greatest reduction. There was no significant difference between the LP and P5 treatment groups. These findings are consistent with the leaf morphological changes shown in Figure 1. In terms of oxidative stress, with the increase of phosphorus concentration, the contents of MDA, H_2_O_2_, and the activity of POD all showed an upward trend. Among them, the contents of MDA and the activity of POD in the P20 and P40 treatment groups were significantly enhanced. The content of H_2_O_2_ in the P40 treatment group was significantly increased, while the activities of SOD and CAT showed a trend of first decreasing, then increasing, and decreasing again. There was a significant improvement in both the LP and HP treatment groups. Compared with CK, there was no significant difference in proline content between the LP and P5 treatment groups; however, P20 and P40 showed significant increases. Inorganic phosphorus (Pi) measurements revealed that Pi content decreased under LP conditions but was significantly elevated in the P5 group. In contrast, no significant differences were detected between the P20, P40, and CK groups. Overall, these results indicate that phosphorus stress influences the physiological status of *P. wrightii* by reducing chlorophyll content, promoting MDA accumulation, and activating the antioxidant enzyme system. However, when phosphorus levels are excessively high, the activities of certain antioxidant enzymes decline, suggesting that high phosphorus stress has a more profound negative impact on plant physiology than low phosphorus stress (Figure 2).

### 2.2. RNA-Seq Analysis of P. wrightii Under Different Phosphorus Stress

#### 2.2.1. Quality Assessment of RNA-Seq Data

To investigate the genetic mechanisms underlying *P. wrightii*’s diverse responses to LP and HP stress, RNA-seq was conducted on the plant leaves. After raw data filtering, we obtained 64.7 GB of clean bases with Q30 scores exceeding 95.85% and GC content ranging between 44.2–47.2% (Table S1). The RNA-seq data can be obtained through accession number PRJNA1335104 (https://www.ncbi.nlm.nih.gov/bioproject/PRJNA1335104 (accessed on 25 September 2025)). De novo assembly was used Trinity software and yielded a total of 149,312 Unigenes (Tables S2 and S3). rincipal component analysis (PCA) of the 15 RNA-seq samples showed that PC1 and PC2 accounted for 19.72% and 11.72% of the total variance, respectively (Figure S1A). Sample correlation heatmap analysis demonstrated high biological reproducibility among replicates (Figure S1B). These results confirm the high quality and reliability of our sequencing data for subsequent bioinformatics analyses.

#### 2.2.2. Identification of Differentially Expressed Genes

**Figure 3:**
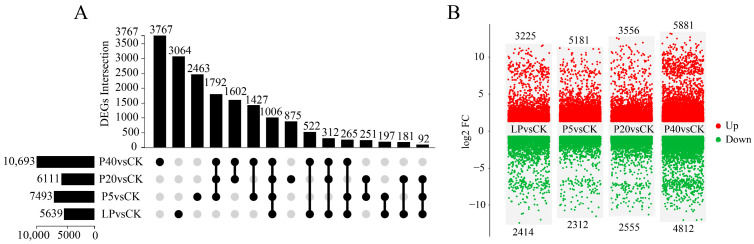
(**A**) UpSet plot of DEGs. The lines connecting the black dots in the Upset plot represent the intersections between sets. The columns and numbers on the left represent the quantity of DEGs contained in each of the four comparison groups. The columns and numbers above show the number of intersection DEGs with the corresponding set below; (**B**) DEGs Volcano map. The numbers in the figure represent the quantity of DEGs, namely CK (0.25 mM), LP (0.025 mM), P5 (5 mM), P20 (20 mM), and P40 (40 mM). From left to right, they are the four comparison groups: LP vs. CK, P5 vs. CK, P20 vs. CK, and P40 vs. CK. The UP (red dot) and Down (green dot) respectively represent the upward and downward adjustment of DEGs, and Log2FC is the corresponding Log2FoldChange value.

Differentially expressed genes (DEGs) were identified by comparing RNA-seq across treatments. A total of 1006 common DEGs were detected across all four comparisons: LP vs. CK, P5 vs. CK, P20 vs. CK, and P40 vs. CK (Figure 3A). Among LP vs. CK, P5 vs. CK, P20 vs. CK, and P40 vs. CK, 5639 (3225 up-regulated and 2414 down-regulated), 7493 (5181 up-regulated and 2312 down-regulated), 6111 (3556 up-regulated and 2555 down-regulated), and 10,693 (5881 up-regulated and 4812 down-regulated) DEGs (Figure 3B). As shown in Figure 3B, the number of DEGs increased markedly with rising phosphorus concentrations. Under HP stress, the number of DEGs was substantially higher than under LP stress. Notably, the number of DEGs in the P40 vs. CK comparison was 1.9 times greater than that in the LP vs. CK comparison. These results suggest that HP stress triggers more extensive transcriptional reprogramming, indicating that these DEGs may play critical roles in the response of *P. wrightii* to varying phosphorus levels.

To further investigate the potential functions of DEGs in the stress response of *P. wrightii*, the Gene Ontology (GO) functional enrichment analysis was performed. The results showed that under different phosphorus treatments, the majority of DEGs were enriched in the biological process (BP) category, primarily involving cellular processes and metabolic processes. In the molecular function (MF) category, DEGs were mainly associated with binding and catalytic activity, while in the cellular component (CC) category, DEGs were enriched in cellular structural components and protein complexes (Figure S2). Although the overall proportion of each GO category remained relatively stable across treatments, the number of DEGs within each category varied considerably, indicating that phosphorus stress induced distinct transcriptional changes.

To identify metabolic pathways affected by phosphorus stress, DEGs were mapped to the Kyoto Encyclopedia of Genes and Genomes (KEGG) database. The top 20 significantly enriched pathways are shown in Figure S3. The metabolic pathways with the most enrichment of DEGs in LP vs. CK are Starch and sucrose metabolism, Nitrogen metabolism, Glycolysis/Gluconeogenesis, Phenylpropanoid biosynthesis, alpha-linolenic acid metabolism, and Photosynthesis antenna proteins. The metabolic pathways with more DEGs enrichment in P5 vs. CK are Photosynthesis, Photosynthesis antenna proteins, Phenylpropanoid biosynthesis, Nitrogen metabolism, Carotenoid biosynthesis, and Plant-pathogen interaction. The metabolic pathways with the most enrichment of DEGs in P20 vs. CK are Phenylpropanoid biosynthesis, Plant-pathogen interaction, Carotenoid biosynthesis, Starch and sucrose metabolism, Porphyrin metabolism, and Flavonoid biosynthesis. The metabolic pathways with the most enrichment of DEGs in P40 vs. CK are Phenylpropanoid biosynthesis, Plant-pathogen interaction, Carotenoid biosynthesis, Starch and sucrose metabolism, photosynthetic antenna proteins, and Nitrogen metabolism. These results suggest that both LP and HP stresses affect key metabolic and defense-related pathways in *P. wrightii*. In particular, pathways related to carbohydrate metabolism, secondary metabolite biosynthesis, photosynthesis, and plant defense responses were strongly influenced, highlighting their potential roles in maintaining phosphorus homeostasis and plant adaptation under stress conditions.

#### 2.2.3. Expression of DEGs in P. wrightii Under Different Phosphorus Stress Conditions

**Figure 4:**
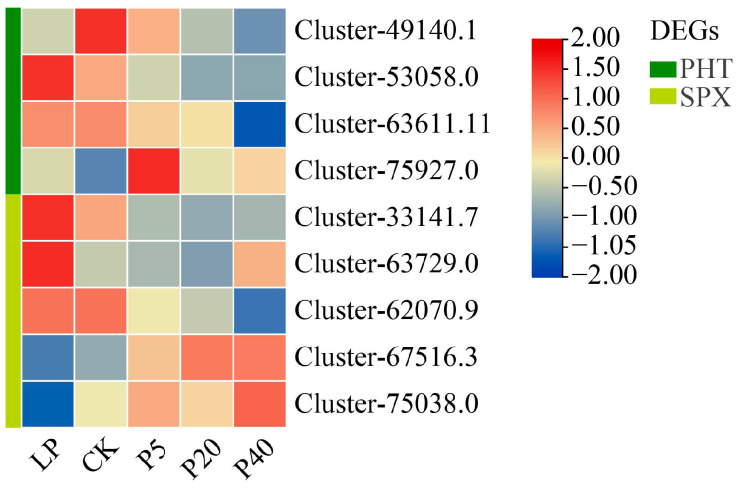
Heat map of DEGs related to phosphorus response. The red and blue boxes represent genes that are upregulated and downregulated under different phosphorus stress conditions. The green and yellow on the left side of the heat map represent the gene grades of the *P. wrighii* PHT family and the SPX gene family respectively. CK (0.25 mM), LP (0.025 mM), P5 (5 mM), P20 (20 mM), and P40 (40 mM). The color scale represents the gene expression levels. During the heat map drawing process, normalization processing is usually carried out, so the legend data is displayed as symmetrically distributed around 0, with red representing high expression and blue representing low expression.

Through bioinformatics analysis, the *P. wrightii* PHT family and SPX gene family were identified to contain 12 and 9 members, respectively. Gene expression levels under different phosphorus treatments are shown in Figure S4A,C. The four treatment groups were respectively compared with the control group. As long as there was a significant expression difference in any of the comparisons of genes, they were defined as DEGs. Otherwise, it is defined as non-DEGs. Based on this criterion, the PHT family included 4 DEGs (2 PHT1 and 2 PHT4), and the SPX family included 5 DEGs (1 SPX, 1 PHO1, 2 SPX-MFS, and 1 NLA). The expression profiles of these 9 DEGs under different phosphorus concentrations are shown in Figure 4. In the PHT family, Cluster-53058.0 and Cluster-75927.0 were upregulated, and Cluster-49140.1 and Cluster-63611.11 were downregulated in LP compared to CK. In P5 vs. CK, P20 vs. CK, and P40 vs. CK, Cluster-49140.1, Cluster-53058.0, and Cluster-63611.11 were consistently downregulated, while Cluster-75927.0 was upregulated. In the SPX family, Cluster-33141.7, Cluster-63729.0, and Cluster-62070.9 were upregulated, whereas Cluster-75038.0 was downregulated in LP vs. CK. For P5 vs. CK, P20 vs. CK, and P40 vs. CK, Cluster-67516.3 and Cluster-75038.0 were upregulated. In addition, a total of 15 members of the PAP (purple acid phosphatase) gene family were identified, including 5 DEGs. Among them, Cluster-45204.0, Cluster-6805.3, Cluster-55786.0, and Cluster-74442.5 were all upregulated in the LP and HP treatment groups, while Cluster-54031.1 was all down-regulated (Figure S4B). All DEGs were predicted and analyzed, and a total of 1169 transcription factors were identified, including 55 families. Among them, WRKY had the most significant number, followed by NAC, AP2/ERF->AP2/ERF-ERF, bZIP, bHLH, C2H2, MYB, and other families (Figure S5). Further analysis of the PHR1 transcription factor family of *P. wrightii* revealed that there was a total of 5 PHR1 and homologous transcription factor PHR-Like members. Among them, two genes were significantly expressed, and Cluster-60971.0 and Cluster-69197.0 were upregulated in LP compared to CK. However, the expressions of P5 vs. CK, P20 vs. CK, and P40 vs. CK were downregulated (Figure S4D). These results indicate that LP and HP stress affect the expression of different gene families in *P. wrightii*, suggesting their roles in multiple aspects of phosphorus stress response.

#### 2.2.4. RT-qPCR Analysis Results

**Figure 5:**
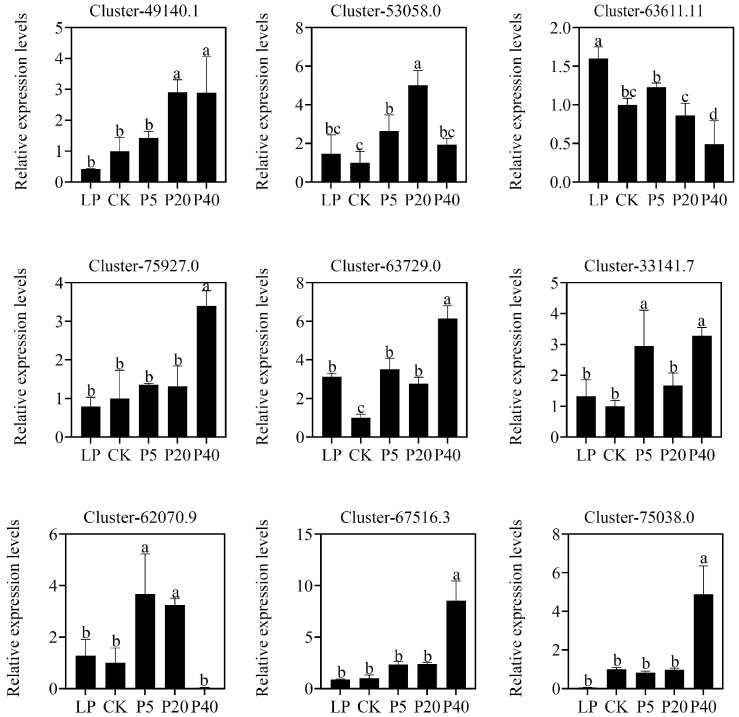
The expression of DEGs in the *P. wrightii* PHT family and SPX family genes under phosphorus stress was analyzed by RT-qPCR. The number directly above each figure is the ID number of DEGs. The standard errors of three biological replications and three technical replications are represented by error bars. CK (0.25 mM), LP (0.025 mM), P5 (5 mM), P20 (20 mM), and P40 (40 mM). The different letters in the figure indicate significant differences among the various groups (*p* < 0.05), and the largest mean was marked with a. The meaning was compared with each of the following means, and those not significantly different were marked with a, until an average was found that was significantly different from it, marked b. Take the maximum average number of marks b as the standard and compare it with the following average number of non-marks, where b is not significant, and c is significant. Take the maximum average number of marks c as the criterion and compare it with the following average number of non-marks, where c is not significant, and d is significant. If two samples have the same letter, the difference is not significant.

To validate the RNA-seq results, 9 DEGs related to phosphorus stress response were selected for quantitative real-time PCR (RT-qPCR) analysis. The RT-qPCR results showed that the expression patterns of these genes were consistent with the FPKM values obtained from RNA-seq, confirming the reliability and accuracy of the RNA-seq data (Figure 5).

### 2.3. Metabolome Analysis of P. wrightii Under Different Phosphorus Stress

#### 2.3.1. Qualitative and Quantitative Analysis of Etabolites

**Figure 6:**
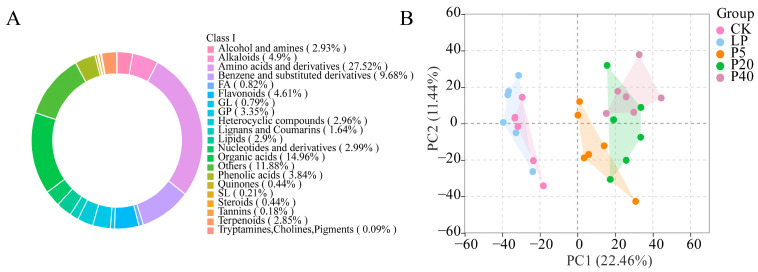
(**A**) Classification diagrams of metabolites identified by *P. wrightii* for different phosphorus treatments. Each color represents a category of metabolites, the area of the color block indicates the proportion of that category, and Class I represents the first-level classification of substances. (**B**) In principal component analysis (PCA), the first principal component (PC1) is represented by the *X*-axis, and the second principal component (PC2) is represented by the *Y*-axis. Groups represent different treatment groups, namely CK (0.25 mM), LP (0.025 mM), P5 (5 mM), P20 (20 mM), and P40 (40 mM).

The leaves of *P. wrightii* were analyzed using the UHPLC-QTOF-MS/MS system, and total ion current (TIC) diagrams of quality control (QC) samples showed highly overlapping curves, indicating high instrument stability and data reliability (Figure S6). A total of 3408 metabolites were detected, which were classified into 21 categories, including 938 amino acids and derivatives, 510 organic acids, 330 benzene and substituted derivatives, 167 alkaloids, 157 flavonoids, 114 glycerophospholipids, 99 lipids, and 405 other compounds (Figure 6A). Principal component analysis (PCA) further revealed the overall metabolic differences among the treatment groups, with PC1 and PC2 contributing 22.46% and 11.44% of the variance, respectively, indicating significant metabolic variation under different phosphorus stress conditions (Figure 6B).

#### 2.3.2. Analysis of Differentially Expressed Metabolites Among Different Treatments

**Figure 7:**
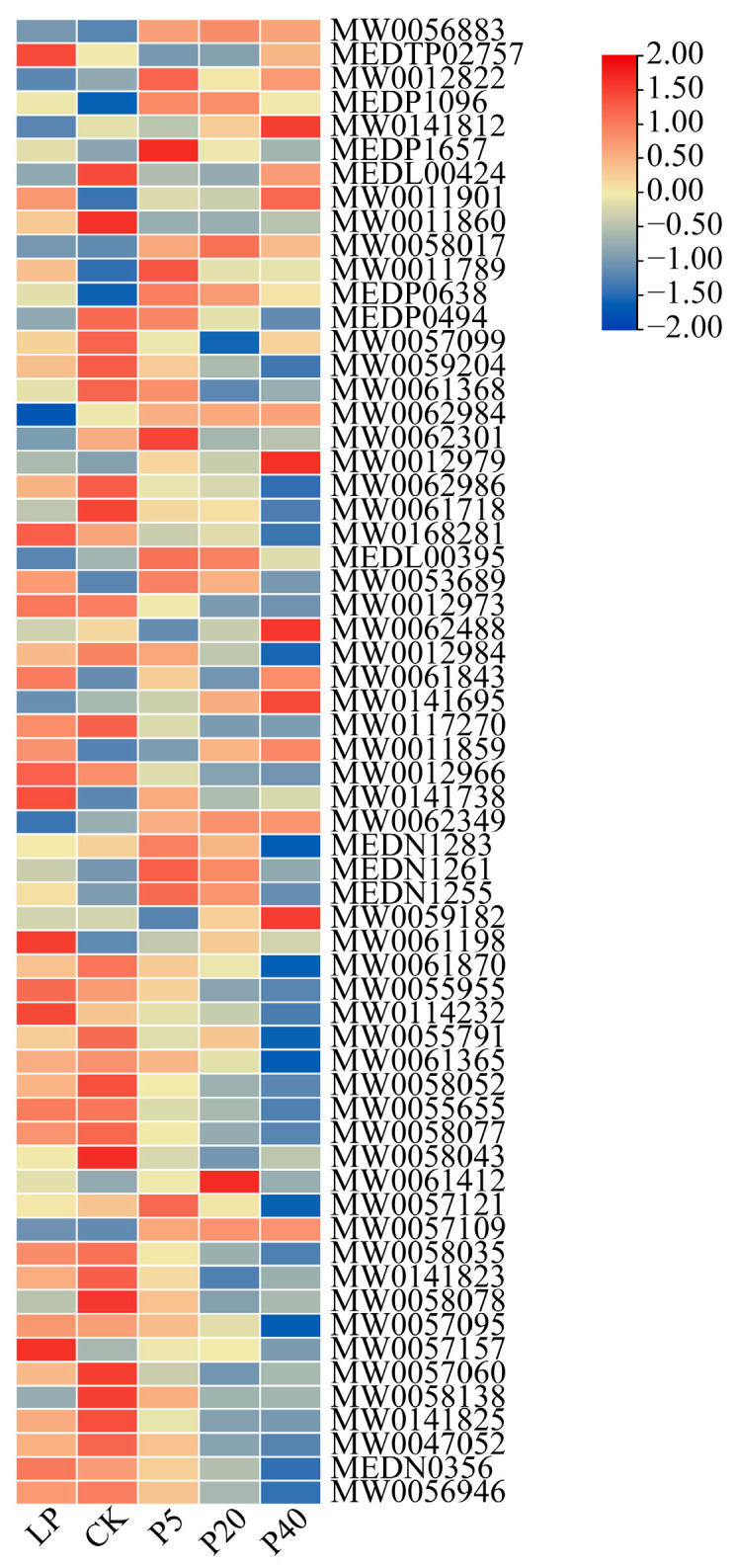
Accumulated heat maps of *P. wrightii* GP under different phosphorus stresses. CK (0.25 mM), LP (0.025 mM), P5 (5 mM), P20 (20 mM), and P40 (40 mM). The red and blue boxes represent DAMs that are up-regulated and down-regulated under different phosphorus stress conditions. The color scale represents the accumulation level of metabolites compared to the color scale. During the heat map drawing process, normalization processing is usually carried out, so the legend data is displayed as symmetrically distributed around 0, with red representing high expression and blue representing low expression.

To identify metabolites that enable *P. wrightii* to adapt to phosphorus stress, differential accumulation of metabolites (DAMs) was analyzed across the Four control groups, revealing a total of 1678 DAMs (Table S4). Volcano plot analysis showed 534 DAMs (121 up-regulated and 413 down-regulated) in LP vs. CK, 450 DAMs (269 up-regulated and 181 down-regulated) in P5 vs. CK, 842 DAMs (409 up-regulated and 433 down-regulated) in P20 vs. CK, and 926 DAMs (327 up-regulated and 599 down-regulated) in P40 vs. CK (Figure S7). KEGG pathway enrichment of these DAMs (*p* ≤ 0.05) highlighted several key metabolic pathways, including general metabolic pathways, biosynthesis of secondary metabolites, arachidonic acid metabolism, pentose phosphate pathway, purine metabolism, glycolysis/gluconeogenesis, glycerolipid metabolism, and glycerophospholipid metabolism (Figure S8). Detailed analysis revealed that most Glycerol phospholipids (GP) contents decreased under high phosphorus (HP) stress, particularly in P40 (Figure 7), whereas flavonoid compounds such as 7-methoxyisoflavone, licoricone, irigenin, dibenzyl ether, and isobavachalcone significantly increased under HP stress. In contrast, eriodyctiol-5,3′-di-O-rutinoside, aloeresin A, and pachyrrhizone increased under low phosphorus (LP) stress (Tables S5 and S6). Given their potent antioxidant properties, flavonoids likely help *P. wrightii* eliminate free radicals and mitigate oxidative damage, suggesting that the differential accumulation of these metabolites under varying phosphorus conditions contributes to the plant’s enhanced adaptability and maintenance of physiological homeostasis.

### 2.4. Combined Transcriptomic and Metabolomic Analysis of the Response Mechanism of P. wrightii to Different Phosphorus Levels

To clarify the phosphorus stress response mechanism of *P. wrightii*, we integrated transcriptomic and metabolomics data. Through KEGG pathway enrichment analysis (Figure S9), we screened out the key pathways co-enriched by DEGs and DAMs, namely Glycerolipid metabolism and Glycerophospholipid metabolism. It is worth noting that the expression trends of some DAMs are not completely consistent with those of related DEGs, which reflects the complex post-transcriptional regulation and metabolic modification between gene transcription and metabolite generation. To further explore the correlation between the two, based on the KEGG database annotations, we identified DEGs encoding the key enzymes in these metabolic pathways and co-mapped them with DAMs to the metabolic pathways, thus constructing a gene–metabolite regulatory network.

In each difference group, genes and metabolites that meet the absolute value of the Pearson correlation coefficient greater than 0.8 and the p-value less than 0.05 are screened and displayed in the nine-quadrant graph (Figure S10). The gene–metabolite interaction network can help understand functional relationships and identify new regulatory elements. The DAMs and DEGs in the Glycerolipid metabolism and Glycerophospholipid metabolism pathways selected and screened were used to draw the gene–metabolite interaction network (Figure S11). No correlation relationship with an absolute value of the Pearson correlation coefficient greater than 0.8 and a p-value less than 0.05 was found between DEGs and DAMs in LP vs. CK. The expression of most DEGs in P5 vs. CK (such as Cluster-37182.0, Cluster-39532.3, Cluster-64735.3, etc.) is similar to that of Citicoline (MEDP1592) and Colfosceril palmitate (MW0056883), 1-Palmitoyl-2-linoleoyl-sn-glycero-3-phosphate (MW0012966) and PC (18:3(6Z,9Z,12Z)/P-16:0) (MW0057109) are related. The four key metabolites were significantly positively correlated with the expression of most DEGs (such as Cluster-39532.3, Cluster-37182.0, Cluster-64735.3, etc.). It was negatively correlated with the expression of most DEGs (such as Cluster-78097.9, Cluster-77096.14 and Cluster-12638.14). In P20 vs. CK, the expression of most DEGs (such as Cluster-61121.0, Cluster-42869.12, Cluster-62070.9, etc.) and Colfosceril palmitate (MW0056883) is associated with 1-Palmitoyl-2-linoleoyl-sn-glycero-3-phosphate (MW0012966). Two key metabolites were significantly positively correlated with the expression of most DEGs (such as Cluster-62070.9, Cluster-37182.0, Cluster-31026.5, etc.). It is negatively correlated with the expression of most DEGs (such as Cluster-72446.3, Cluster-67702.3 and Cluster-67516.3). On P40vs. The expression of most DEGs in CK (such as Cluster-62241.0, Cluster-51434.5, Cluster-77293.7, etc.) is similar to that of Choline (MEDP0125), Diethanolamine (MEDP0128), and PC(18:3, 6Z,9 Related to Z,12Z)/P-16:0 (MW0057109), Colfosceril palmitate (MW0056883), and 1-Palmitoyl-2-linoleoyl-sn-glycero-3-phosphate (MW0012966) The five key metabolites were significantly positively correlated with the expression of most DEGs (such as Cluster-51434.5, Cluster-62070.9, Cluster-37182.0, etc.). It was negatively correlated with the expression of most DEGs (such as Cluster-67702.3, Cluster-62241.0 and Cluster-67516.3). It is worth noting that, as can be found from Figure 5, the Cluster-67516.3 and Cluster-62070.9 genes involved in the Glycerolipid metabolism pathway are members of the SPX domain gene family.

**Figure 8:**
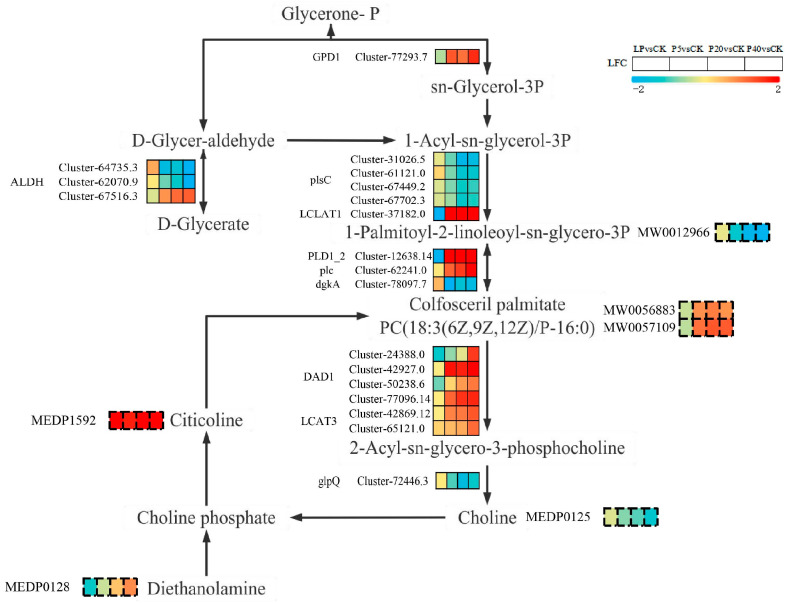
Response mechanisms of the *P. wrightii* Glycerolipid metabolism and Glycerophospholipid metabolism pathways under phosphorus stress. DEGs and DAMs are respectively represented by solid and dashed boxes in the path, with the boxes from left to right being LP vs. CK, P5 vs. CK, P20 vs. CK, and P40 vs. CK, and LFC being the corresponding Log2FoldChange value. Red and blue, respectively, represent the upward and downward adjustments of DEGs and DAMs. CK (0.25 mM), LP (0.025 mM), P5 (5 mM), P20 (20 mM), and P40 (40 mM). plsC:1-acyl-sn-glycerol-3-phosphate acyltransferase [EC:2.3.1.51]; GPD1:glycerol-3-phosphate dehydrogenase (NAD+) [EC:1.1.1.8]; LCLAT1:lysocardiolipin and lysophospholipid acyltransferase [EC:2.3.1.- 2.3.1.51]; PLD1-2:phospholipase D1/2 [EC:3.1.4.4]; plc:phospholipase C [EC:3.1.4.3]; dgkA:diacylglycerol kinase (ATP) [EC:2.7.1.107]; DAD1:phospholipase A1 [EC:3.1.1.32]; LCAT3:phospholipase A1 [EC:3.1.1.32]; qlpQ:glycerophosphoryl diester phosphodiesterase [EC:3.1.4.46]; ALDH:aldehyde dehydrogenase (NAD+) [EC:1.2.1.3].

To comprehensively evaluate the potential molecular mechanisms of *P. wrightii* under phosphorus stress, we plotted the Glycerolipid metabolism and Glycerophospholipid metabolism pathways by analyzing the KEGG pathways of DEGs and DAMs (Figure 7). We noticed that Citicoline accumulated similarities in the four control groups. Colfosceril palmitate, Diethanolamine, and PC (18:3(6Z,9Z,12Z)/P-16:0) accumulated increased in P5 vs. CK, P20 vs. CK, and P40 vs. CK, while accumulated decreased in LP vs. CK. The accumulation of Choline and 1-Palmitoyl-2-linoleoyl-sn-glycero-3-phosphate increased in LP vs. CK, while the accumulation decreased in P5 vs. CK, P20 vs. CK, and P40 vs. CK. In addition, compared with LP vs. CK, more DEGs and DAMs in P5 vs. CK, P20 vs. CK and P40 vs. CK were significantly upregulated. The above results indicate that the expression of specific genes and the accumulation of metabolites under the same metabolic pathway, as well as the regulation of specific metabolic pathways, lead to the differences in *P. wrightii*’s responses to low phosphorus and high phosphorus stress (Figure 8).

## 3. Discussion

Research on *P. rightii*’s phosphorus removal and absorption has been reported [36,37], but the molecular mechanism of its response to phosphorus stress remains unknown. The morphological manifestations of plants vary with different phosphorus treatments. Low phosphorus stress inhibits the photosynthetic activity of plants, resulting in a slower metabolism [38]. High phosphorus stress leads to the loss of leaf greenness and phosphorus poisoning in plants [7], thereby affecting plant growth and development, which is consistent with the results of this study. Under high phosphorus stress, compared with the control group, the leaves of *P. wrightii* turned yellow and the chlorophyll content decreased significantly, while there was no significant difference under low phosphorus stress. Reactive oxygen species (ROS) are key signaling molecules that enable cells to respond rapidly to various stimuli [39]. This study found that under high phosphorus stress (P40) conditions, the contents of MDA and H_2_O_2_ in plant leaves accumulated significantly. This result clearly indicates that phosphorus stress disrupts the dynamic balance between the production and clearance of reactive oxygen species (ROS) within plants, leading to a significant accumulation of ROS and subsequently triggering severe oxidative stress, which damages the structural and functional integrity of cell membranes. To cope with oxidative stress, plants activate their defense systems through the use of antioxidant enzymes. In this experiment, the enzymatic activity of POD increased with the rise in phosphorus concentration. In contrast, the dynamic changes in SOD and CAT activities were more complex, exhibiting a trend of first decreasing, then increasing, and then decreasing again with increasing phosphorus concentration. This result reflects the different antioxidant strategies that plants may adopt under different degrees of phosphorus stress to maintain cellular homeostasis. Under high phosphorus stress, plants accumulate a large amount of proline. Proline accumulation usually enhances the tolerance to osmotic stress, maintains the water absorption capacity of cells, and thereby alleviates osmotic dysregulation caused by stress [40]. The results of inorganic phosphorus detection in plant leaves showed that the inorganic phosphorus content in the LP treatment group decreased, while that in the P5 group significantly increased, indicating that LP stress limited phosphorus absorption. In contrast, moderate phosphorus increase (P5) promoted phosphorus accumulation. However, there was no difference between the P20 and P40 groups and CK. Considering their growth inhibition and changes in the antioxidant system, it is speculated that high phosphorus may cause metabolic disorders, leading to a decrease in phosphorus absorption and utilization efficiency. Overall, the negative impact of HP stress on plants is more severe than that of LP stress, as evidenced by a reduction in chlorophyll content, the induction of reactive oxygen species accumulation, and membrane lipid peroxidation, ultimately leading to oxidative damage in cells. In response, plants enhance their ROS clearance capacity by increasing the activity of certain antioxidant enzymes and accumulating substances such as proline to stabilize the intracellular environment, thereby forming a complex defense network against phosphorus stress.

Maintaining the homeostasis of phosphorus within plant cells is an important biological mechanism, and phosphate transporters (PHT) play a key role in Pi acquisition and in vivo balance [41]. Based on protein sequences, structural features, subcellular localization and functions, the PHT family is divided into four subfamilies: PHT1 (Sugar (and other) transporter), PHT2 (Phosphate transporter), PHT3 (Mitochndrial carrier pretein), and PHT4 (Major Facility (Superfamily) [42]. In model plants such as *Arabidopsis thaliana* and *Oryza sativa*, most high-affinity *PHT* genes are predominantly expressed in roots, although some can also be induced in leaves under Pi starvation [43,44]. In *O. sativa*, *OsPHT2* and *OsPHT6* are primarily expressed in roots, but their expression increases in leaves during phosphorus (Pi) deficiency [45]. Based on phylogenetic, sequence, and functional analyses, the *PHT* gene family of *P. wrightii* was identified, and the expression of its differentially expressed genes (DEGs) under low phosphorus (LP) and high phosphorus (HP) stress was analyzed. Some members of the PHT1 and PHT4 subfamilies were upregulated under LP stress and downregulated under HP stress, whereas other members exhibited the opposite trend. The SPX domain encoding protein plays a significant role in Pi homeostasis and signaling pathways. Proteins containing the SPX domain are classified into four families based on whether other domains exist in their structure: SPX (SPX-Single domain), SPX-MFS (SPX-Major Facility Superfamily) (also known as VPTs or PHT5) SPX-EXS (SPX-ERD1/XPR1/SYG1) (also known as PHO1) and SPX-RING (SPX-really Interesting New Gene) (also called NLA) [46]. In *A. thaliana* and *O. sativa*, most *SPX* genes are Pi-responsive, except *OsSPX4* and AtSPX4 [47]. *NLA*, *OsSPX-MFS1*, and *OsSPX-MFS3* are repressed under Pi deficiency, while *OsSPX-MFS2* is induced [46,48]. In *P. wrightii*, members of the SPX and PHO1 subfamilies were upregulated under LP treatment and downregulated under HP treatment, while NLA subfamily members showed downregulation under LP and upregulation under HP. The two DEGs of SPX-MFS members exhibited opposite expression patterns, consistent with observations in *O. sativa*. PAP not only release phosphorus from the soil but also regulate its distribution throughout plant growth [49]. In this study, five PAP family DEGs were identified, of which four were upregulated under both LP and HP treatments, while one was downregulated, suggesting potential functional diversification. AtPHR1 is a MYB transcription factor that plays a key role in Pi starvation signaling [50]. PHR1 affects the expression of PSI genes, including key components of Pi signaling such as SPX, PHT, PAP, miRNA399s, and IPS1 [51]. Two PHR-like genes in *P. wrightii* were upregulated under LP stress but downregulated under HP stress, indicating their potential role in regulating SPX, *PHT*, and *PAP* to maintain phosphorus homeostasis. Collectively, these genes play key roles in the plant’s adaptation to varying phosphorus conditions, facilitating phosphorus uptake, transport, and physiological balance.

The cell membrane is mainly composed of lipids, proteins and carbohydrates, among which the phospholipid bilayer is its basic framework. The growth, development and physiological and biochemical functions of plants can affect the structure and composition of membrane lipids [52]. Phosphorus stress induces the expression of plant phospholipases (such as PLC and PLD), accelerating Phosphatidylcholine (PC) and Phosphatidylethanolamine (PE) is hydrolyzed into Phosphatidate (PA) and diacylglycerol (DAG), and then DAG is used as the substrate. Under the catalysis of enzyme transformation sulfoquinovosyldiacylglycerol (SQDG) [53]. Pi reduces the relative amount of total phosphatide, hunger and added some of phospholipids, especially monogalactosyldiacylglycerol (MGDG), digalactosyldiacylglycerol (DGDG) and (SQDG) [54]. Ding et al. [55] determined that in the presence of both phosphorus deficiency and excess, the synthesis of phosphorylated metabolites was reduced. *P. wrightii* responds to changes in phosphorus nutritional status by precisely regulating the turnover of membrane phospholipids. We observed that under low phosphorus stress, the expression of phospholipase-coding genes (such as PLD1-2, plc, DAD1, LCAT3) was down-regulated, and the corresponding degradation products The accumulation levels of Colfosceril palmitate and PC (18:3(6Z,9Z,12Z)/P-16:0) also decreased simultaneously. On the contrary, under high-phosphorus conditions, the expression of these genes and metabolites is upregulated. This synergistic change in the expression of genes and metabolites indicates that *P. wrightii* may maintain the integrity of the cell membrane structure by inhibiting the degradation of membrane phospholipids under low phosphorus stress. Under high phosphorus stress, the degradation pathway is activated to release inorganic phosphorus for other life activities. In addition, PC can be hydrolyzed by DAD1 and LCAT3 to form lysophospholipids. glpQ, as an important hydrolase in the phospholipid metabolic pathway, is responsible for catalyzing the hydrolysis of glycerophosphate diesters to generate glycerophosphate and corresponding alcohols (such as choline), which are used for the next round of phospholipid synthesis (such as PC). In this study, the expression of glpQ was upregulated under low phosphorus stress and downregulated under high phosphorus stress. Therefore, glpQ is involved in *P. wrightii*’s adaptation to phosphorus stress and plant growth and development. SQDG is widely present in plant chloroplasts, participates in the function and evolution of photosynthetic membranes, and is of great significance to plant photosynthesis [56]. However, there was no significant difference in the content of SQDG in *P. wrightii* under different phosphorus stresses. Therefore, SQDG may not play a major role in the photosynthesis, growth or development of *P. wrightii*, which is basically consistent with the research of Yu et al. And wang et al. [8,57]. It is worth noting that in our study, it was found that some genes encoding the ALDH enzyme are members of the SPX domain gene family. This gene may play a key role in regulating the Glycerolipid metabolism and Glycerophospholipid metabolism pathways. In particular, it affects the activity of related enzymes by responding to the homeostasis of phosphorus within cells. The above-mentioned genes require further functional verification.

## 4. Materials and Methods

### 4.1. Materials and Sample Preparation

*P. wrighti* seedlings were collected from the Huaxi River, Guiyang City, Guizhou Province, China (106°27′ E, 26°11′ N) and propagated under laboratory conditions. The plants were cultivated in a controlled light incubator at 24 ± 2 °C with a 12-h light/12-h dark photoperiod (Figure S12). The phosphorus stress test selected newly propagated plants after two weeks of culture, with a plant length of approximately 35 to 40 cm. For the experiment, seedlings were placed in transparent columns (15 cm in diameter and 50 cm in height), each containing 6.5 L of either the standard 10% solution or a phosphorus-deficient Hoagland nutrient solutions (Hunan Hoagland Biotechnology Co., Ltd., Xiangtan, China). Six seedlings were placed in each column. After a 7—day acclimation period, phosphorus treatments were applied using solutions with different phosphorus concentrations: CK (control, 0.25 mM), LP (low phosphorus, 0.025 mM), and HP (high phosphorus, 5 mM, 20 mM, and 40 mM), with the pH adjusted to 6.0. Each treatment included three independent biological replicates. The nutrient solution was refreshed once during the treatment period. After 72 h of exposure, plants were harvested and rinsed thoroughly three times with sterile water to remove residual nutrient solutions. The leaves were then immediately frozen in liquid nitrogen and stored at −80 °C for subsequent analyses.

### 4.2. Determination of Chlorophyll, Antioxidant Indicators, and Phosphorus Content in Plant Leaves

Chlorophyll content was measured using a SPAD-502 Plus chlorophyll meter (Konica Minolta, Tokyo, Japan). The activities of antioxidant enzymes in plant leaves, including superoxide dismutase (SOD), peroxidase (POD), and catalase (CAT), as well as the content of malondialdehyde (MDA), Hydrogen Peroxide (H_2_O_2_), Proline (Pro), inorganic phosphorus content (Pi) were determined using commercially available kits (Sod No: BC0170, Pod No:BC0090, Cat No:BC0200, Mda No:BC0025, H_2_O_2_ No:BC3590, Pro No:BC0290, and Pi No:BC2840; Solarbio Science & Technology Co., Ltd., Beijing, China) following the manufacturer’s instructions and previous reports [58,59,60,61,62]. Each treatment group included three independent biological replicates.

### 4.3. Leaf RNA-Seq and Data Analysis

A reference genome-free RNA-seq strategy was adopted [13]. RNA extraction, quality assessment, library construction, and high-throughput sequencing were performed by Metware Biotechnology Co., Ltd. (Wuhan, China), with three independent biological replicates per treatment group. Sequencing was conducted on the Illumina NovaSeq^TM^ X Plus platform (Illumina, CA, USA) using the sequencing-by-synthesis method.

The original data was filtered using fastp 0.23.2 [63], the clean reads were assembled using Trinity v2.15.1 [64], and the CDS of the assembled transcripts was predicted using TransDecoder v5.3.0 [65]. Obtain the amino acid sequence corresponding to the transcript. The Unigene sequence was compared with the KEGG, NR, Pfam, Swiss-Prot, GO, COG/KOG and Trembl databases using DIAMOND v2.0. [66] After predicting the amino acid sequence of Unigene, the HMMER 3.2 [67] package software was used to compare it with the Pfam database to obtain the annotation information of Unigene. The parameters of the seven major databases are shown in Table S7. The expression levels of transcripts were calculated using RSEM v1.3.1 software [68], and then the FPKM of each transcript was calculated based on its length. According to the expression levels of each sample, DESeq2 v1.22.2 software [69,70] was used to conduct differential analysis of gene expression. Based on the corrected P-values, If the log2Fold Change is greater than or equal to 1 and the FDR is less than 0.05, the genes are screened as significantly differentially expressed genes (DEGs). Upset [71] of DEGs analysis figure and volcanic figure drawing through online website https://cnsknowall.com/#/Home/Contain/BottomContainAll (accessed on 7 November 2025) and https://www.bioinformatics.com.cn/ (accessed on 6 June 2025). The heat map was drawn after normalizing the DEGs data using TBtools-II v2.136. software [72]. The software parameters are shown in Table S8.

### 4.4. Leaf Metabolome and Data Analysis

Metabolome analysis was conducted by Metware Biotechnology Co., Ltd. (Wuhan, China), with six independent biological replicates in each group. The analysis was conducted using a TripleTOF 6600+ mass spectrometer (SCIEX, Foster City, CA, USA) and an LC-30A ultra-high performance liquid chromatograph (Shimadzu, Kyoto, Japan), namely an ultra-high performance liquid chromatography-tandem mass spectrometry (UPLC-MS/MS) system. The raw data were converted to mzXML format using ProteoWizard [73]. Peak extraction, alignment, and retention time correction were carried out using the XCMS program. Principal component analysis (PCA) was conducted with the prcomp function in R v4.1.2 [74,75] to assess the overall variation and grouping of samples. The orthogonal partial least squares discriminant analysis (OPLS-DA) model was applied to identify key metabolites, and the variable importance in projection (VIP) score was calculated to evaluate the contribution of each metabolite to sample classification. Differentially accumulated metabolites (DAMs) were screened based on the criteria of VIP > 1 and |log_2_ fold change| ≥ 1. Identified metabolites were functionally annotated using the Kyoto Encyclopedia of Genes and Genomes (KEGG) database [76], and then mapped to KEGG pathways to explore metabolic changes associated with phosphorus stress. The heat map was drawn after normalizing the DAMs data using TBtools-II v2.136 software.

### 4.5. Combined RNA-Seq and Metabolome Analyses

The Pearson correlation coefficient between genes and metabolites was calculated using the cor function of R v2.2.5. Use Rv3.3.6 and R v1.3.4 to draw the nine-quadrant graph and network graph for correlation analysis. Based on the Kyoto Encyclopedia of Genes and Genomes (KEGG) database (https://www.genome.jp/kegg/ (accessed on 11 November 2025)), correlation analysis was conducted between differentially expressed genes (DEGs) and differentially accumulated metabolites (DAMs) within the same metabolic pathways. The correlated DEGs and DAMs were then mapped to KEGG pathway charts to reveal their functional relationships. Final visualizations were created using Microsoft Excel 2021 and Microsoft PowerPoint 2021 (Microsoft, Redmond, WA, USA).

### 4.6. Analysis of the Expression Characteristics of Key Genes in the Phosphorus Pathway by RT-qPCR

To verify the reliability of RNA-seq data, nine genes related to phosphate transporters were selected from DEGs for RT-qPCR analysis. The primers are listed in Table S9. The relative expression levels of each gene were calculated using the 2^−ΔΔCt^ method [77].

## 5. Conclusions

This study elucidated the physiological and molecular regulatory mechanisms underlying the response of *P. wrightii* to phosphorus stress. Under HP stress, plants exhibited visible leaf yellowing and a marked decrease in chlorophyll content, while no significant changes were observed under LP stress. In terms of oxidative stress, phosphorus stress induces the accumulation of reactive oxygen species, as evidenced by a significant increase in the contents of MDA and H_2_O_2_, accompanied by the activation of the antioxidant defense system. The activities of POD, SOD, and CAT all increased, with SOD and CAT displaying a dynamic pattern of initially increasing and then decreasing under HP stress. At the molecular level, several DEGs belonging to the PHT, SPX, and PAP gene families, as well as the transcription factor *PHR1*, were identified and analyzed under varying P conditions. Through joint analysis, the study revealed the response differences of 6 DAMs and 19 DEGs to the *P. wrightii* Glycerolipid metabolism and Glycerophospholipid metabolism pathways under different phosphorus stresses. Overall, this research deepens our understanding of the mechanisms by which submerged plants respond to phosphorus stress, providing a theoretical foundation for ecological restoration and eutrophication management in shallow lake ecosystems.

## Data Availability

The RNA-seq data presented in this study are openly available; the processed data can be obtained through accession number PRJNA1335104 (https://www.ncbi.nlm.nih.gov/bioproject/PRJNA1335104 (accessed on 25 September 2025)). Accession numbers for DEGs: PwACTIN, PwNLA, PwPHO1, PwPHT1, PwPHT1-Like, PwPHT4, PwPHT4-Like, PwSPX, PwSPX-MFS, PwSPX-MFS-Like: C_AA126357.1—C_AA126366.1.

## Supplementary Materials

The following supporting information can be downloaded at https://www.mdpi.com/article/10.3390/plants14233556/s1, Figure S1: (A) Sample PCA, the first principal component (PC1) is represented by the *X*-axis, and the second principal component (PC2) is represented by the *Y*-axis. (B) Sample correlation heatmap, Pearson correlation coefficient (R^2^) > 0.8 between biological duplicate samples; Figure S2: GO Function Annotation of *P. wrightii* DEGs under phosphorus stress. (A) LP vs. CK, (B) P5 vs. CK, (C) P20 vs. CK, (D) P40 vs. CK. The horizontal axis represents the secondary GO entry, and the vertical axis represents the number of genes on the comment of this GO entry; Figure S3: KEGG enrichment analysis of *P. wrightii* DEGs under phosphorus stress. (A) LP vs. CK, (B) P5 vs. CK, (C) P20 vs. CK, (D) P40 vs. CK. The vertical axis represents the KEGG pathway, and the horizontal axis represents the Rich factor. The greater the Rich factor, the greater the degree of enrichment; Figure S4: Expression heat maps of members of the (A) PHT family, (B) PAP gene family, (C) SPX gene family, and (D) PHR1 transcription factor family. LP (0.025 mM), CK (0.25 mM), P5 (5 mM), P20 (20 mM), P40 (40 mM). Red and blue represent genes that are upregulated and downregulated under different phosphorus stresses; Figure S5: Analysis of the *P. wrightii* transcription factor family under phosphorus stress; Figure S6. (A): The total ion flow (TIC) shows the positive ion modes, (B): The total ion flow (TIC) shows the negative ion modes. Figure S7: Volcanic map of *P. wrightii* DAMs under phosphorus stress. (A) LP vs. CK, (B) P5 vs. CK, (C) P20 vs. CK, (D) P40 vs. CK. The green dots represent the down-regulated differential metabolites, the red dots represent the up-regulated differential metabolites, and the gray dots represent the metabolites detected but with insignificant differences. The horizontal axis represents the logarithm of the multiple of the relative content difference in a certain metabolite in the two groups of samples, and the vertical axis represents the level of significance of the difference; Figure S8: KEGG enrichment analysis of *P*. *wrightii* DAMs under phosphorus stress. (A) LP vs. CK, (B) P5 vs. CK, (C) P20 vs. CK, (D) P40 vs. CK. The vertical axis represents the KEGG pathway, and the horizontal axis represents the Rich factor. The greater the Rich factor, the greater the degree of enrichment.; Figure S9: Bar chart of *P. wrightii* KEGG enrichment analysis under phosphorus stress. (A) LP vs. CK, (B) P5 vs. CK, (C) P20 vs. CK, (D) P40 vs. CK. The horizontal axis represents the P-value of the significance test enriched to this pathway, and the red and green axes represent the metabolome and RNA-seq respectively; Figure S10: Correlation analysis and nine-quadrant chart. (A) LP vs. CK, (B) P5 vs. CK, (C) P20 vs. CK, (D) P40 vs. CK. Each point represents a pair of correlations, with the horizontal axis representing the Log2FC of genes and the vertical axis representing the Log2FC of metabolites. The black dotted line is divided into quadrants 1–9 from left to right and from topto bottom. For quadrant 5, the genes, metabolites, and differently grouped genes and metabolites are not differentially expressed. For quadrants 3 and 7, the genes and metabolites have the same differential expression patterns. For genes and metabolites with consistent expression level trends, the genes may positively regulate the changes in the metabolites. For quadrants 1 and 9, the genes and metabolites have the opposite differential expression patterns. For genes and metabolites with inconsistent expression level trends, the genes may negatively regulate the changes in the metabolites. For quadrants 2, 4, 6, 8, when the metabolites are unchanged, the genes are upregulated or downregulated and when the genes are unchanged, the metabolites are upregulated or downregulated; Figure S11: The gene–metabolite interaction network diagrams of the Glycerolipid metabolism and Glycerophospholipid metabolism pathways. In the figure, metabolites are marked with green squares and genes with red circles. Solid lines represent positive correlation, while dashed lines represent negative correlation. ko00561 represents the Glycerolipid metabolism pathway and ko00564 represents the Glycerophospholipid metabolism pathway. Figure S12: Diagram of the experimental setup; Table S1: RNA-seq data of 15 samples; Table S2: Assembly Result Statistics Table; Table S3: Annotation statistics table of each database; Table S4: According to the statistics table of DAMs for the four processing groups; Table S5: DAMs statistics Table of the four processing group GP; Table S6: Accumulation of flavonoid DAMs in LP vs. CK, P5 vs. CK, P20 vs. CK, and P40 vs. CK; Table S7: Software and parameters of the gene function annotation database; Table S8: Analyze the list of software and their versions; Table S9: Primers used for RT-qPCR analysis in this study.
