# Supplementary material for: Integrating RNA-Seq and Metabolomic Perspectives Reveals the Mechanism of Response to Phosphorus Stress of Potamogeton wrightii
Source: Plants (Basel). 2025 Nov 21;14(23):3556. doi: 10.3390/plants14233556 (PMC12693802; doi:10.3390/plants14233556)
Supplement: Supplementary file 1 [file plants-14-03556-s001.zip › Supplementary Figure S1.pdf]

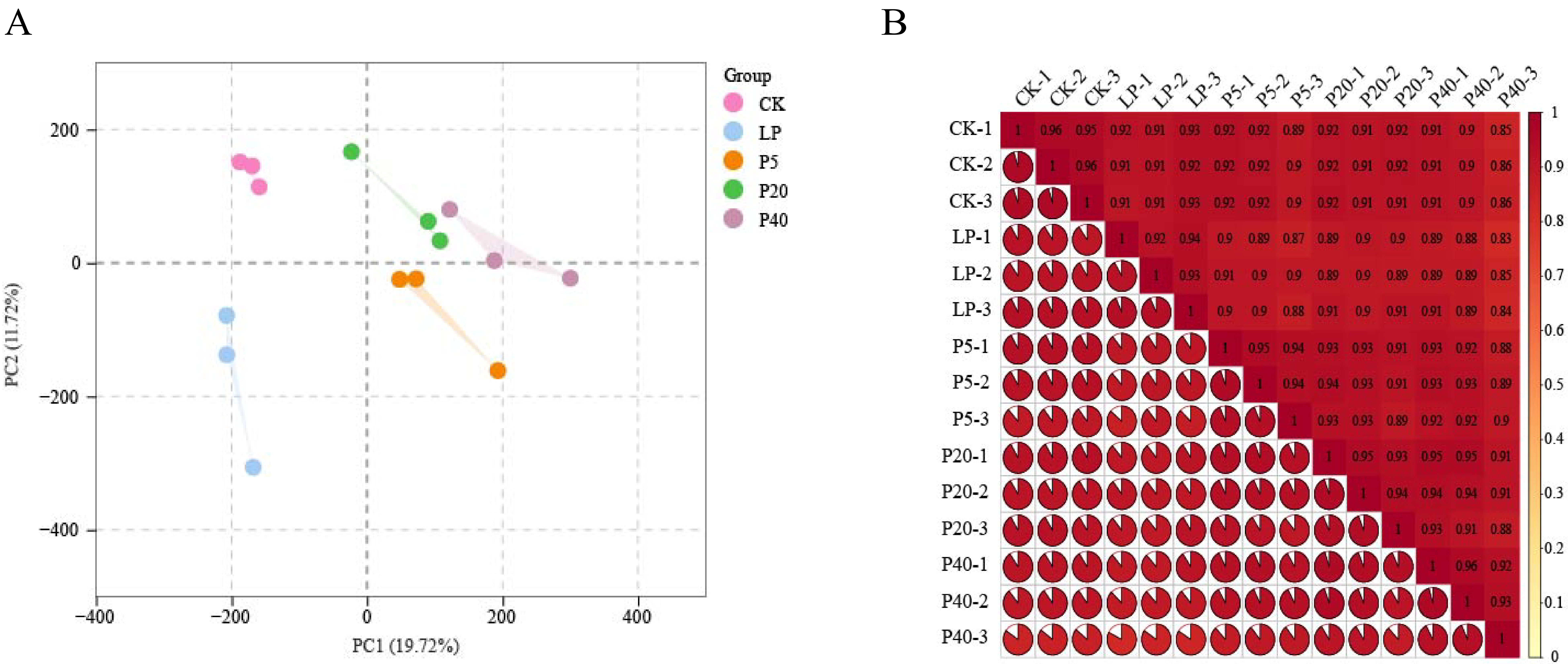

Figure S1.(A) Sample PCA analysis, the first principal component (PC1) is represented by the X-axis, and the second principal component (PC2) is represented by the Y-axis.(B) Sample correlation heatmap, Pearson correlation coefficient (R^2)> 0.8 between biological duplicate samples.
