# Supplementary material for: Integrating RNA-Seq and Metabolomic Perspectives Reveals the Mechanism of Response to Phosphorus Stress of Potamogeton wrightii
Source: Plants (Basel). 2025 Nov 21;14(23):3556. doi: 10.3390/plants14233556 (PMC12693802; doi:10.3390/plants14233556)
Supplement: Supplementary file 1 [file plants-14-03556-s001.zip › Supplementary Figure S2.pdf]

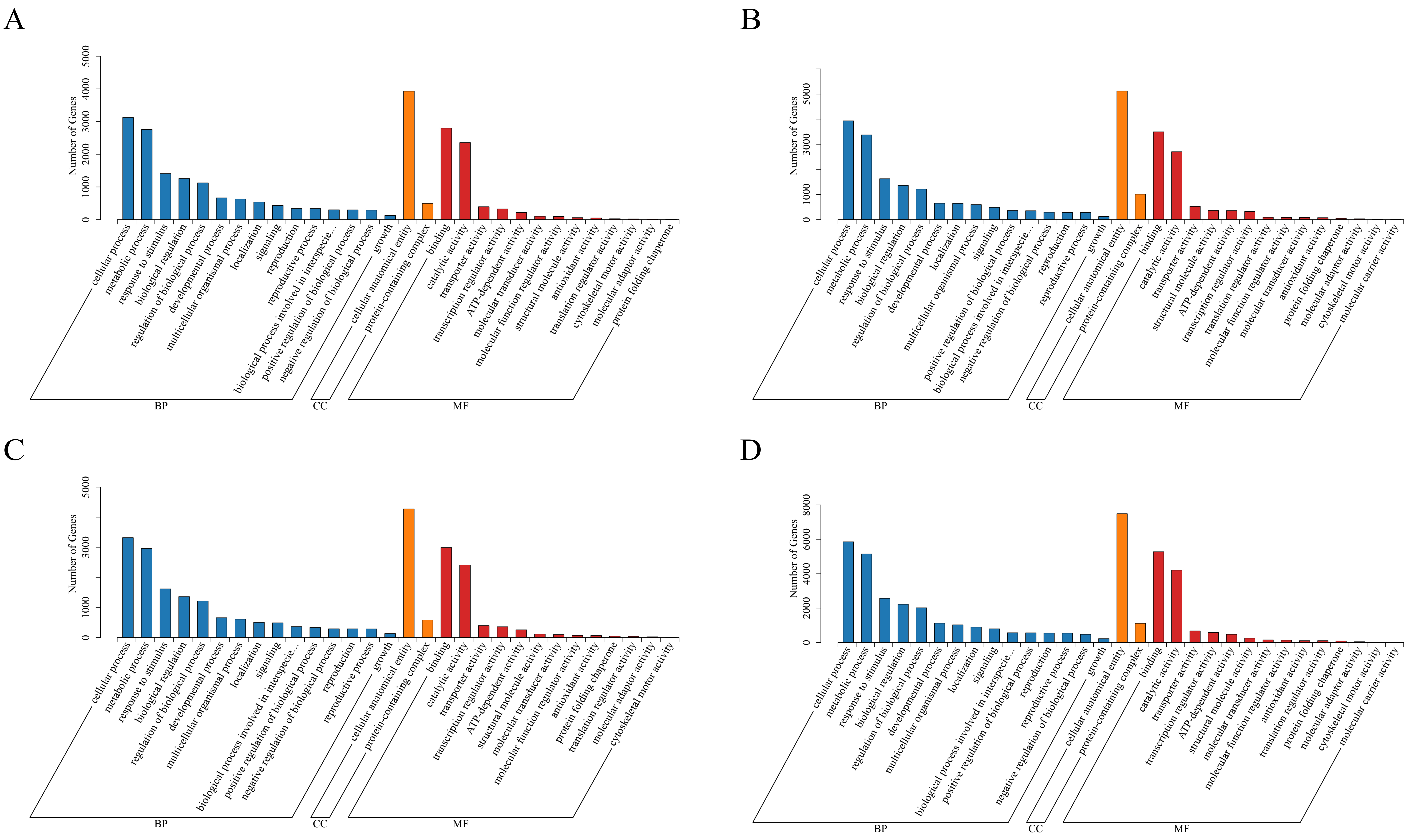

Figure S2. GO Function Annotation of *P.wrightii* DEGs under phosphorus stress. (A) LP vs. CK, (B) P5 vs. CK, (C) P20 vs. CK, (D) P40 vs. CK. The horizontal axis represents the secondary GO entry, and the vertical axis represents the number of genes on the comment of this GO entry.
