# Supplementary material for: Integrating RNA-Seq and Metabolomic Perspectives Reveals the Mechanism of Response to Phosphorus Stress of Potamogeton wrightii
Source: Plants (Basel). 2025 Nov 21;14(23):3556. doi: 10.3390/plants14233556 (PMC12693802; doi:10.3390/plants14233556)
Supplement: Supplementary file 1 [file plants-14-03556-s001.zip › Supplementary Figure S3.pdf]

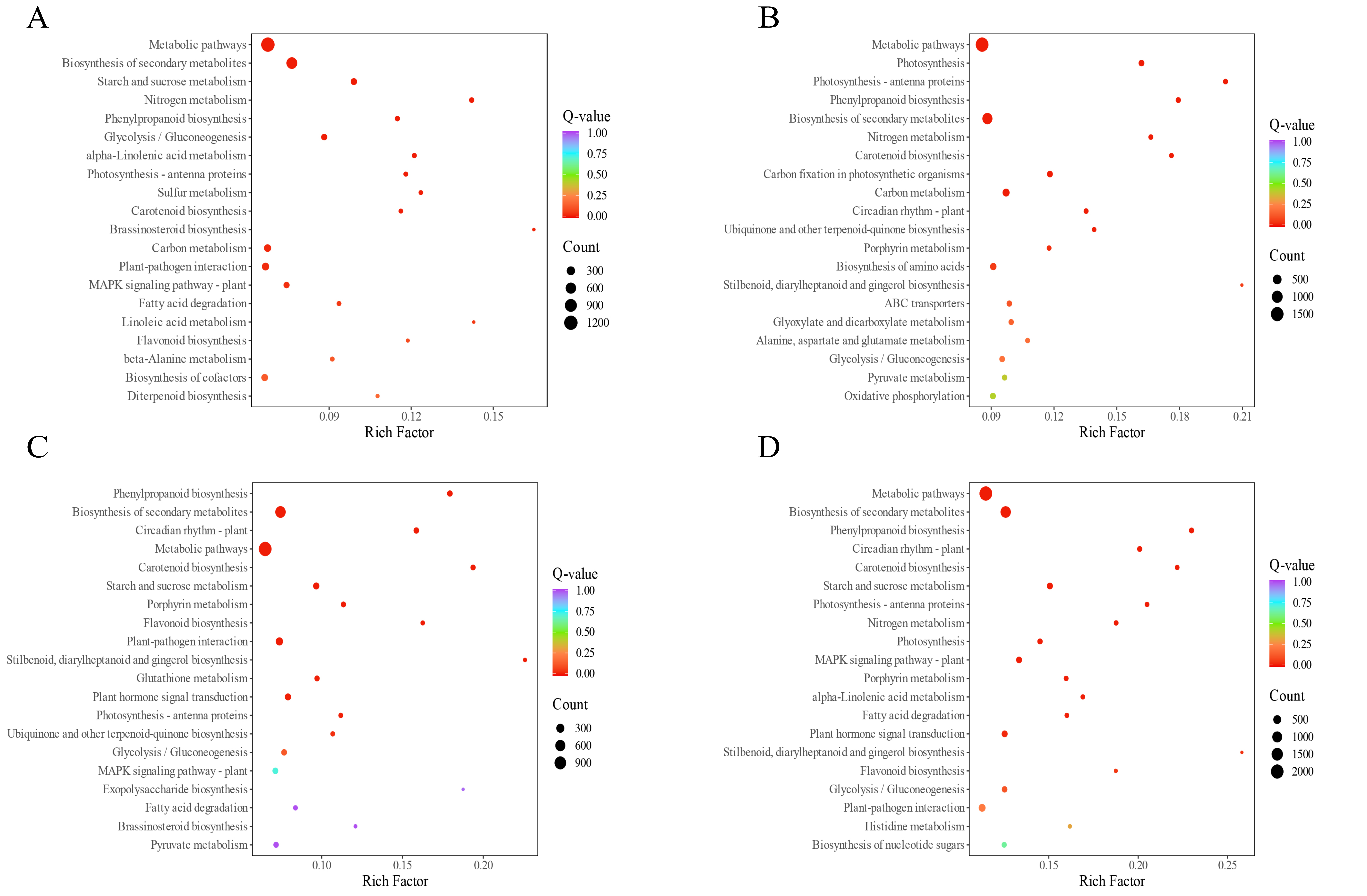

Figure S3. KEGG enrichment analysis of *P. wrightii* DEGs under phosphorus stress. (A) LP vs. CK, (B) P5 vs. CK, (C) P20 vs. CK, (D) P40 vs. CK. The vertical axis represents the KEGG pathway, and the horizontal axis represents the Rich factor. The greater the Rich factor, the greater the degree of enrichment.
