# Supplementary material for: Integrating RNA-Seq and Metabolomic Perspectives Reveals the Mechanism of Response to Phosphorus Stress of Potamogeton wrightii
Source: Plants (Basel). 2025 Nov 21;14(23):3556. doi: 10.3390/plants14233556 (PMC12693802; doi:10.3390/plants14233556)
Supplement: Supplementary file 1 [file plants-14-03556-s001.zip › Supplementary Figure S4.pdf]

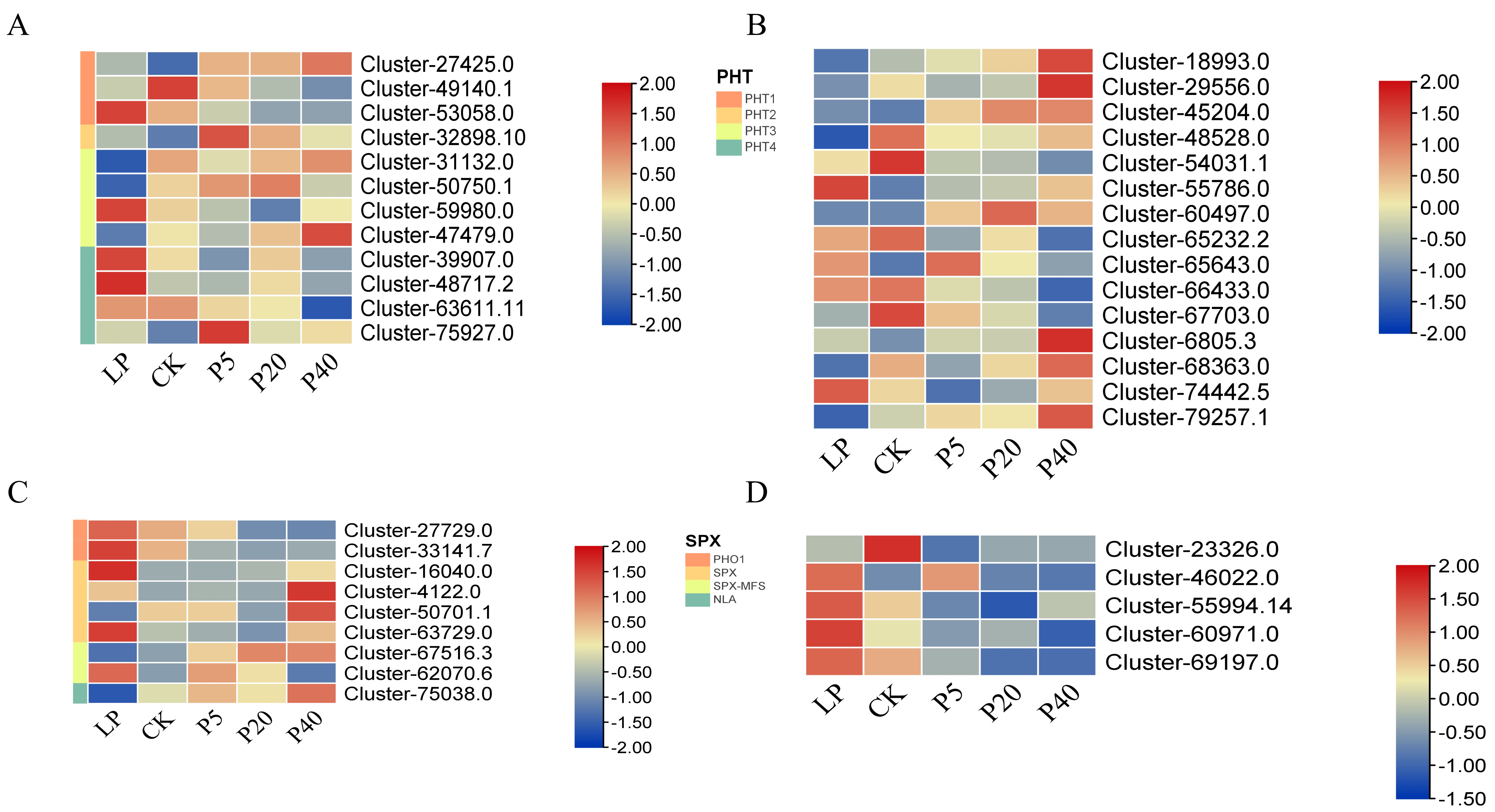

Figure S4. Expression heat maps of members of the (A)PHT family, (B)PAP gene family, (C)SPX gene family, and (D) PHR1 transcription factor family. LP (0.025 mM), CK(0.25mM),P5(5mM), P20(20mM), P40 (40mM). Red and blue represent genes that are upregulated and downregulated under different phosphorus stresses.
