# Supplementary material for: Integrating RNA-Seq and Metabolomic Perspectives Reveals the Mechanism of Response to Phosphorus Stress of Potamogeton wrightii
Source: Plants (Basel). 2025 Nov 21;14(23):3556. doi: 10.3390/plants14233556 (PMC12693802; doi:10.3390/plants14233556)
Supplement: Supplementary file 1 [file plants-14-03556-s001.zip › Supplementary Figure S7.pdf]

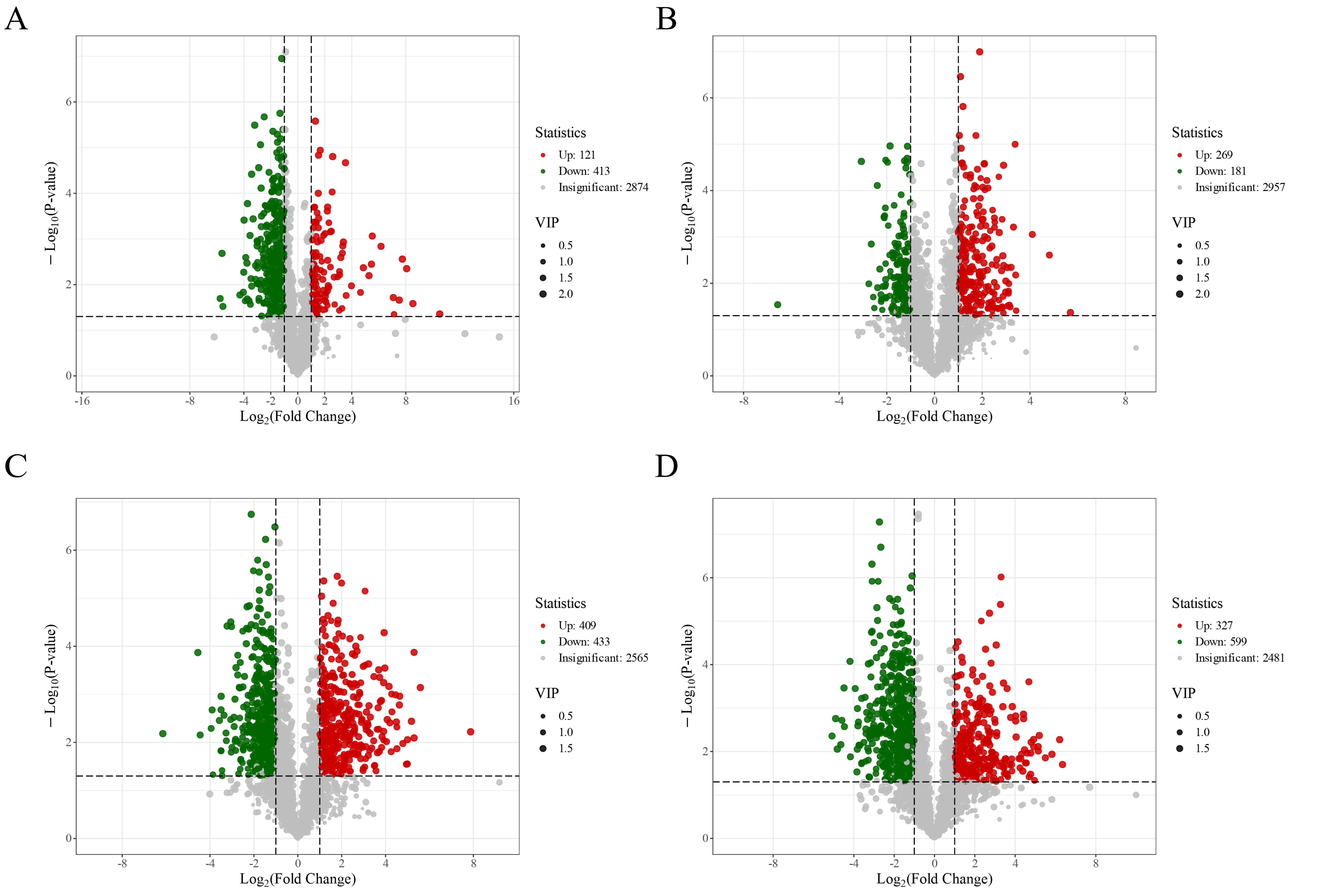

Figure S7. Volcanic map of *P.wrightii* DAMs under phosphorus stress. (A) LP vs. CK, (B) P5 vs. CK, (C) P20 vs. CK, (D) P40 vs. CK. The green dots represent the down-regulated differential metabolites, the red dots represent the up-regulated differential metabolites, and the gray dots represent the metabolites detected but with insignificant differences. The horizontal axis represents the logarithm of the multiple of the relative content difference of a certain metabolite in the two groups of samples, and the vertical axis represents the level of significance of the difference.
