# Supplementary material for: Integrating RNA-Seq and Metabolomic Perspectives Reveals the Mechanism of Response to Phosphorus Stress of Potamogeton wrightii
Source: Plants (Basel). 2025 Nov 21;14(23):3556. doi: 10.3390/plants14233556 (PMC12693802; doi:10.3390/plants14233556)
Supplement: Supplementary file 1 [file plants-14-03556-s001.zip › Supplementary Figure S9.pdf]

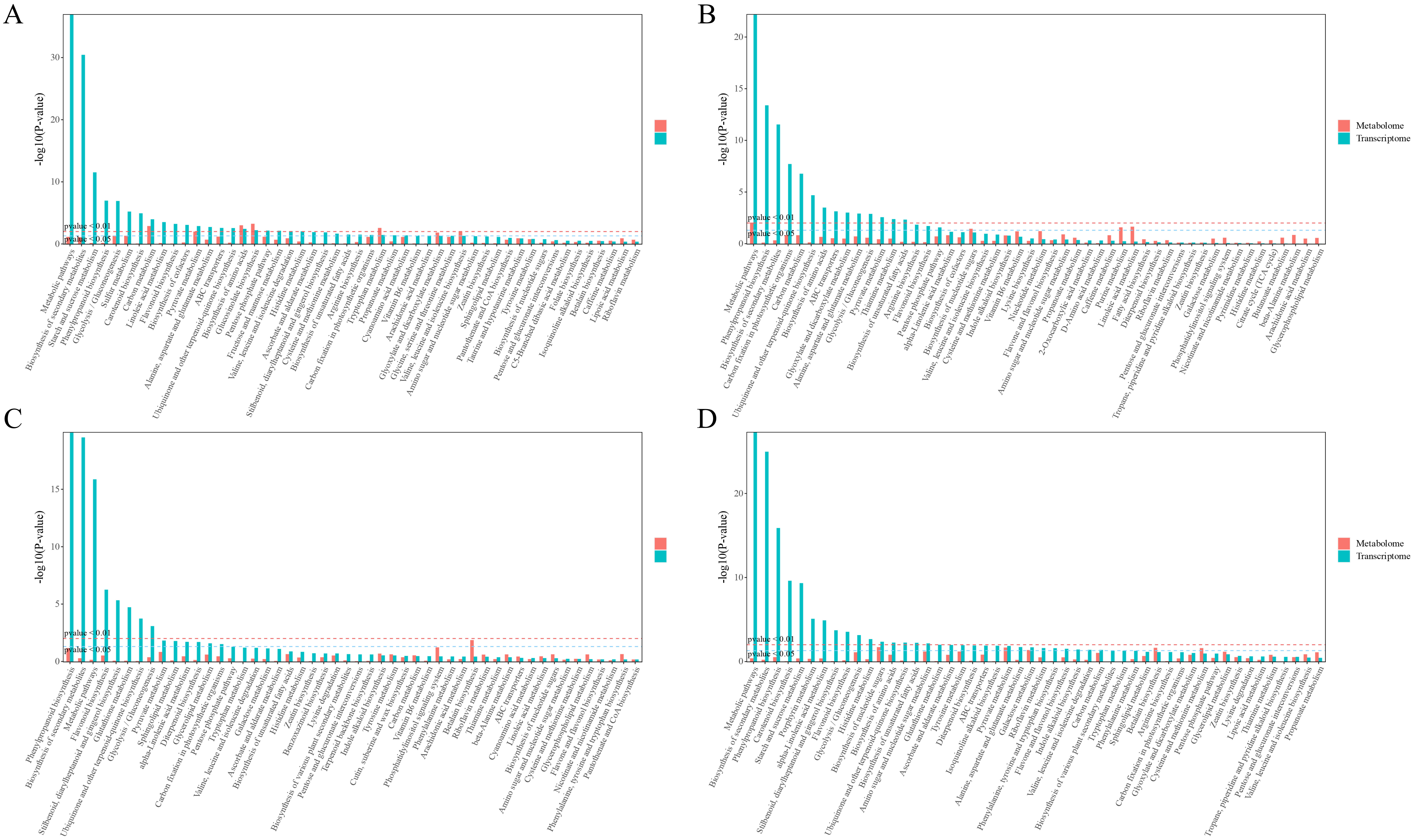

Figure S9. KEGG enrichment bar chart of *P.wrightii* DEGs under phosphorus stress. (A) LP vs. CK, (B) P5 vs. CK, (C) P20 vs. CK, (D) P40 vs. CK. The horizontal axis represents the name of the KEGGpathway, the vertical axis represents the P-value of the significance test enriched to this pathway, and red and green represent the metabolome and transcriptome, respectively.
