# Supplementary material for: Integrating RNA-Seq and Metabolomic Perspectives Reveals the Mechanism of Response to Phosphorus Stress of Potamogeton wrightii
Source: Plants (Basel). 2025 Nov 21;14(23):3556. doi: 10.3390/plants14233556 (PMC12693802; doi:10.3390/plants14233556)
Supplement: Supplementary file 1 [file plants-14-03556-s001.zip › Supplementary Figure S10.pdf]

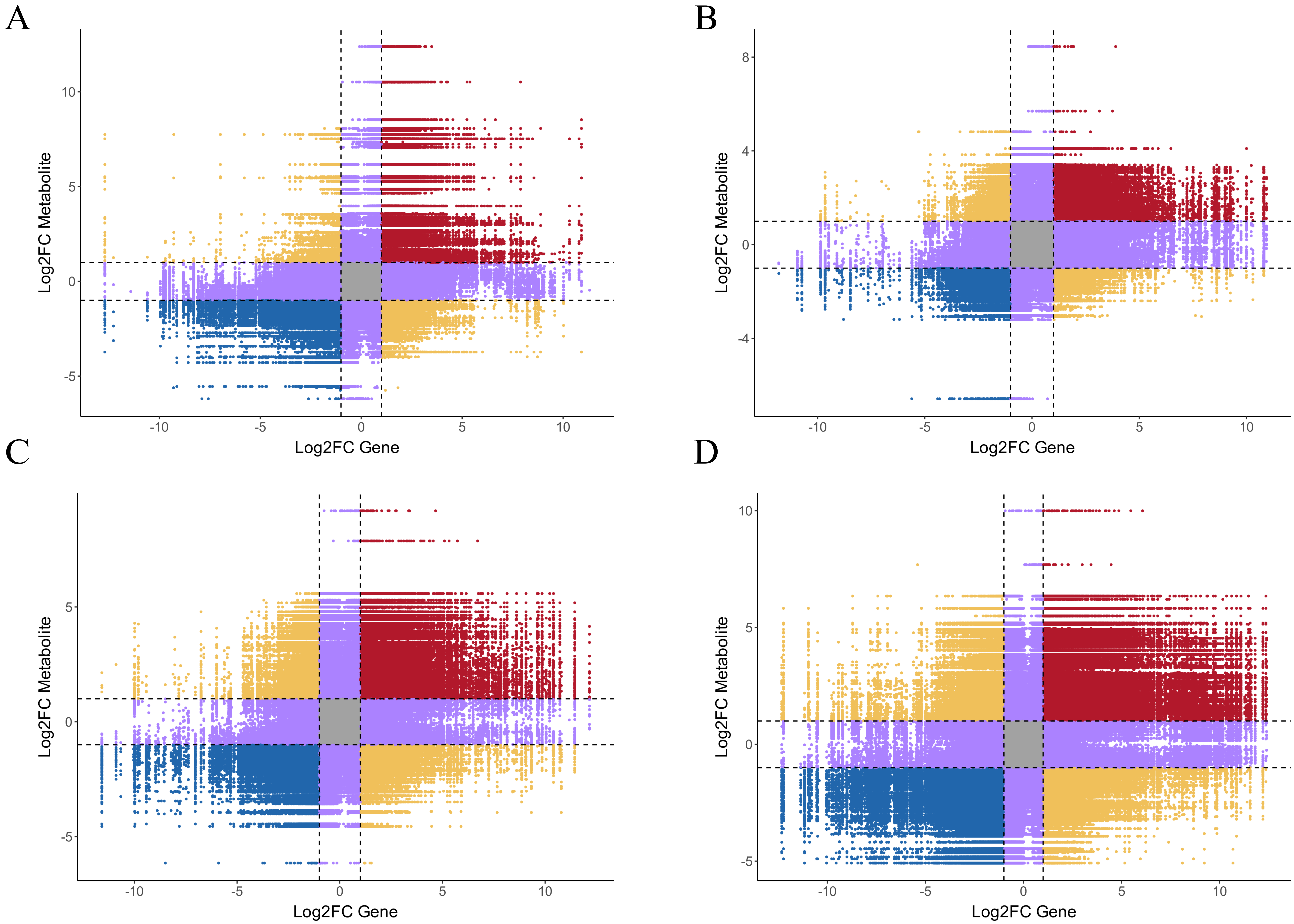

Figure S10. Correlation analysis and nine-quadrant chart. A: LP vs. CK, B: P5 vs. CK, C: P20 vs. CK, D: P40 vs. CK.Each point represents a pair of correlations, with the horizontal axis representing the Log2FC of genes and the vertical axis representing the Log2FC of metabolitesThe black dotted line is divided intoquadrants 1–9 from left to right and from topto bottom.For quadrant 5, the genes, metabolites, and differently grouped genes and metabolites are not differentially expressed. For quadrants 3 and 7,the genes and metabolites have the same differential expression patterns. For genes and metabolites with consistent expression level trends, the genes may positively regulate the changes in the metabolites. For quadrants 1 and 9, the genesand metabolites have the opposite differential expression patterns. For genes and metabolites with inconsistent expression level trends, the genes may negatively regulate the changes in the metabolites. For quadrants 2, 4, 6, 8, when the metabolites are unchanged, the genes are upregulated or downregulated and when the genes are unchanged, the metabolites are upregulated or downregulated.
