# Supplementary material for: Integrating RNA-Seq and Metabolomic Perspectives Reveals the Mechanism of Response to Phosphorus Stress of Potamogeton wrightii
Source: Plants (Basel). 2025 Nov 21;14(23):3556. doi: 10.3390/plants14233556 (PMC12693802; doi:10.3390/plants14233556)
Supplement: Supplementary file 1 [file plants-14-03556-s001.zip › Supplementary Figure S11.pdf]

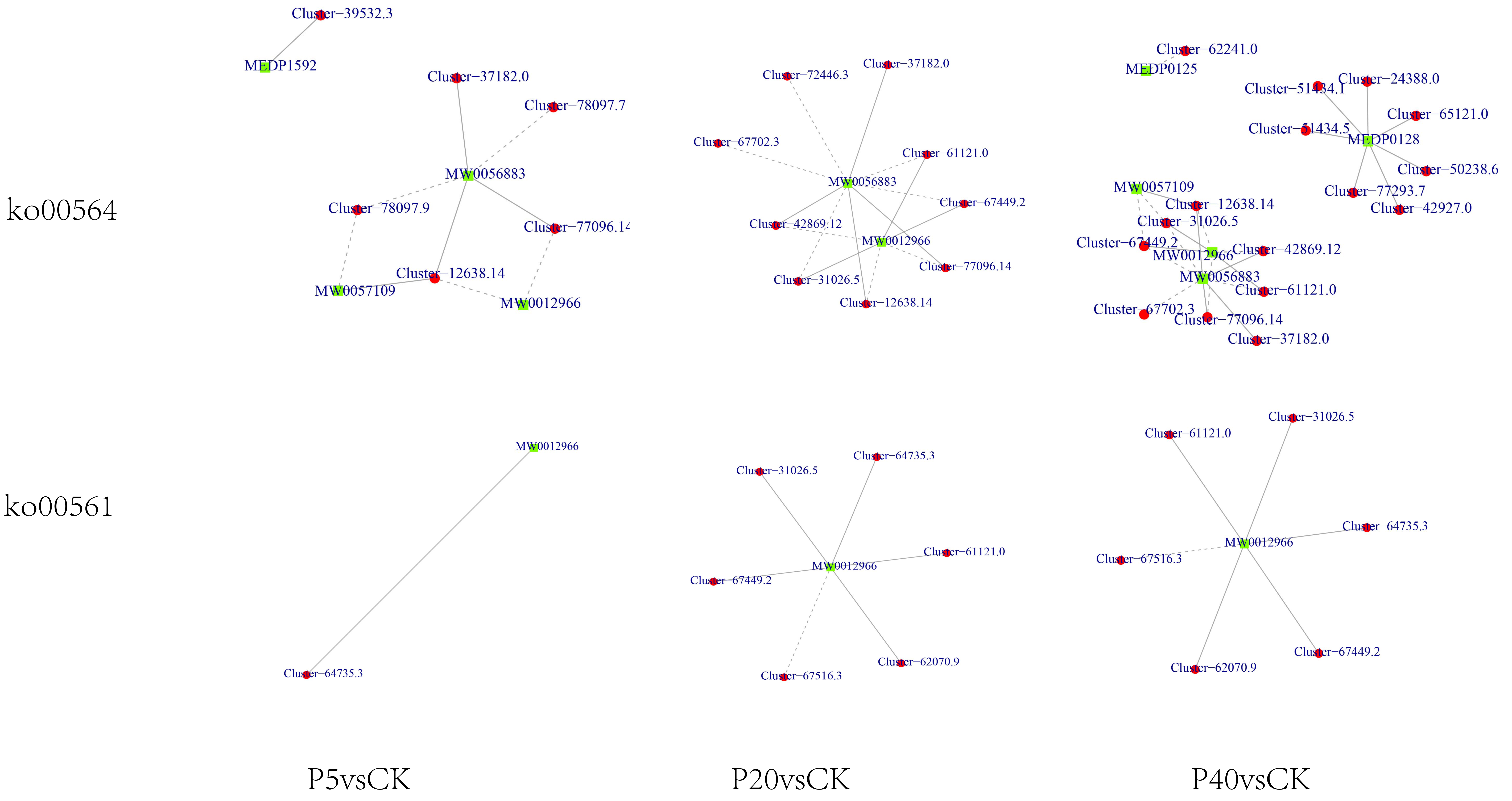

Figure S11. The gene-metabolite interaction network diagrams of the Glycerolipid metabolism and Glycerophospholipid metabolism pathways. In the figure, metabolites are marked with green squares and genes with red circles. Solid lines represent positive correlation, while dashed linesrepresent negative correlation. ko00561 represents the Glycerolipid metabolism pathway and ko00564 represents the Glycerophospholipid meta-bolism pathway.
