# Supplementary figures and images for: Integrating RNA-Seq and Metabolomic Perspectives Reveals the Mechanism of Response to Phosphorus Stress of Potamogeton wrightii
Source: Plants (Basel). 2025 Nov 21;14(23):3556. doi: 10.3390/plants14233556 (PMC12693802; doi:10.3390/plants14233556)

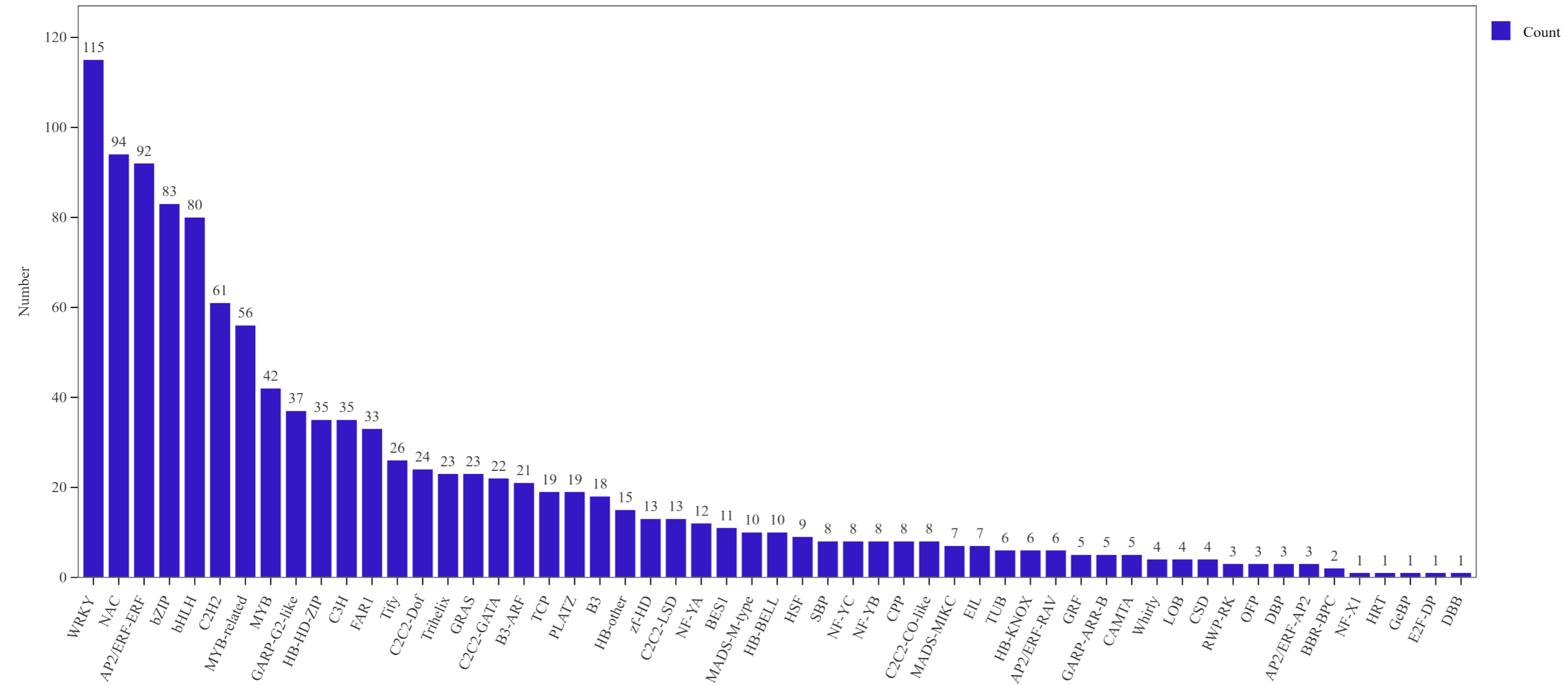

Figure S5. Analysis of the *P.wrightii* transcription factor family under phosphorus stress.

Supplement: Supplementary file 1 [file plants-14-03556-s001.zip › Supplementary Figure S5.pdf]

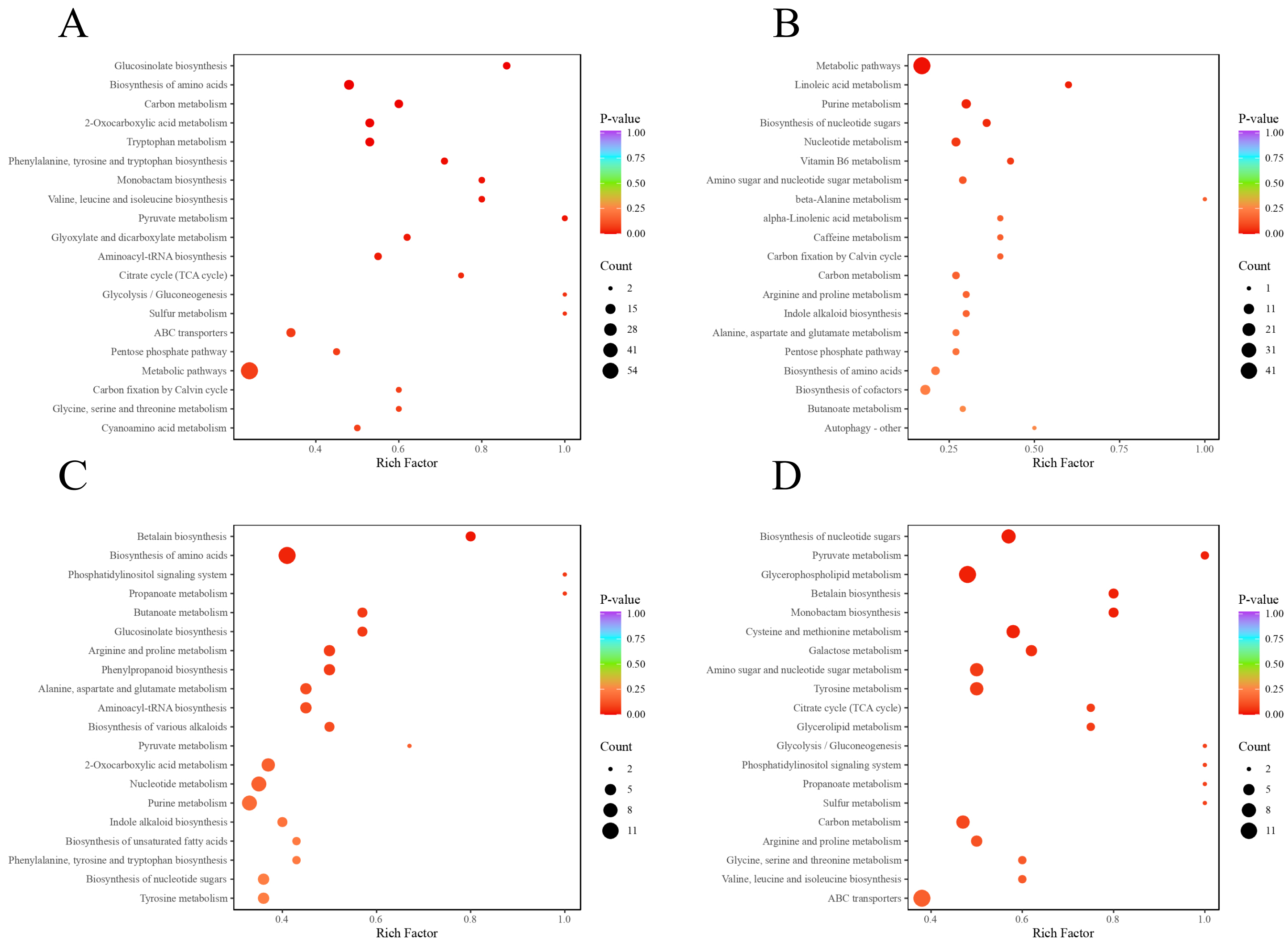

Supplement: Supplementary file 1 [file plants-14-03556-s001.zip › Supplementary Figure S8.pdf]

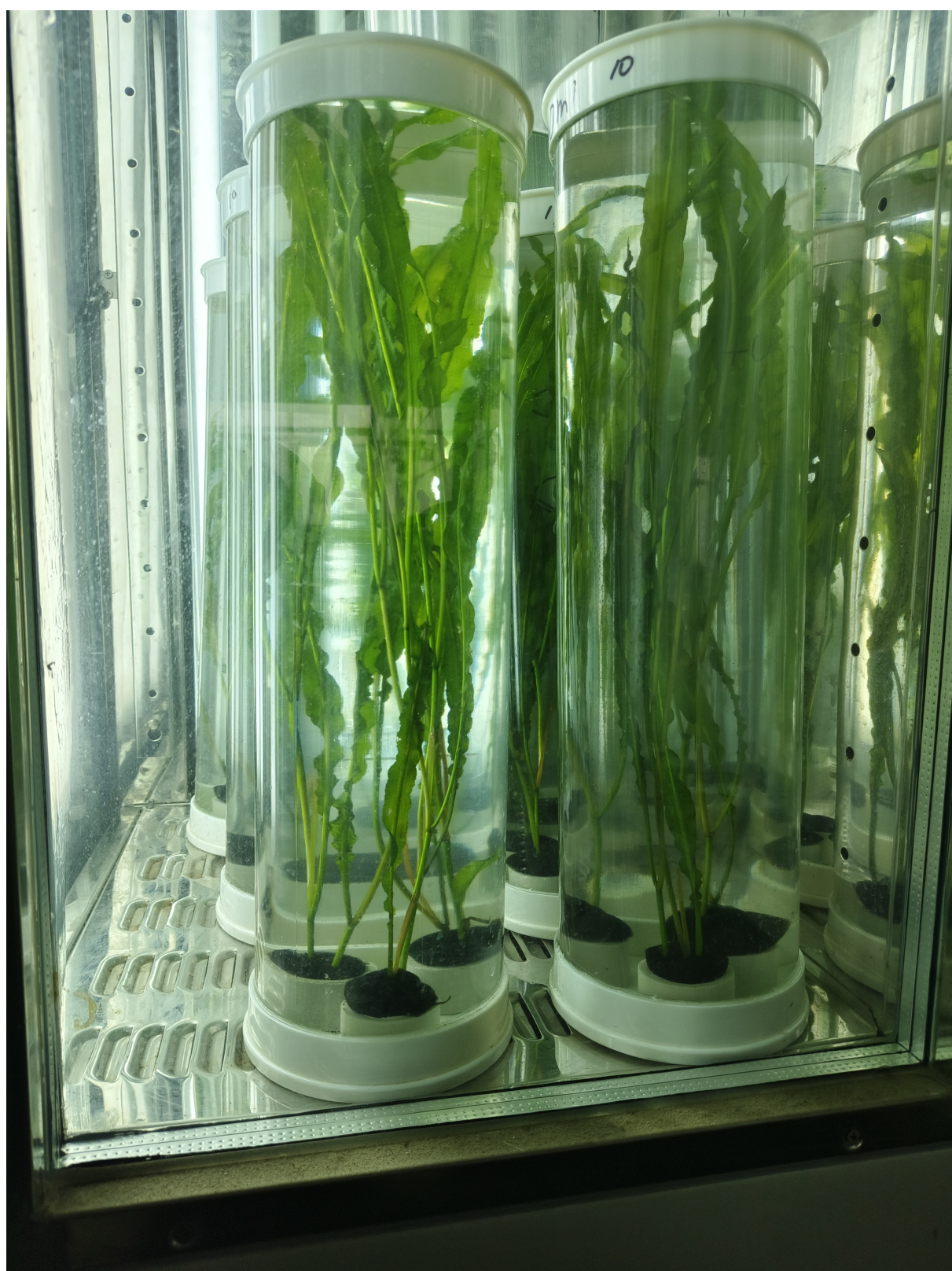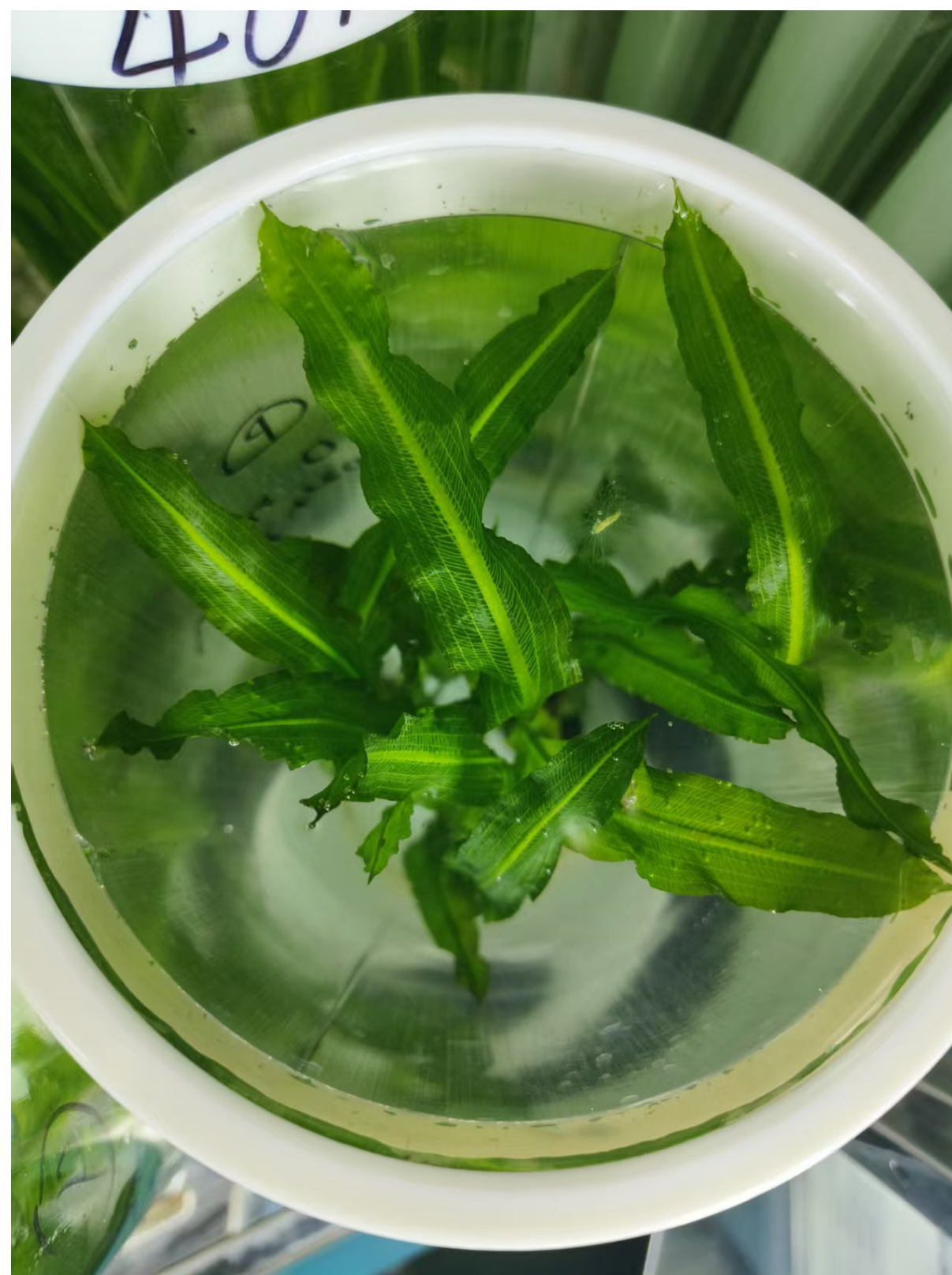

Figure S12. Diagram of the experimental setup.

Supplement: Supplementary file 1 [file plants-14-03556-s001.zip › Supplementary Figure S12.pdf]
